# An immune-based biomarker signature is associated with mortality in COVID-19 patients

**DOI:** 10.1172/jci.insight.144455

**Published:** 2021-01-11

**Authors:** Michael S. Abers, Ottavia M. Delmonte, Emily E. Ricotta, Jonathan Fintzi, Danielle L. Fink, Adriana A. Almeida de Jesus, Kol A. Zarember, Sara Alehashemi, Vasileios Oikonomou, Jigar V. Desai, Scott W. Canna, Bita Shakoory, Kerry Dobbs, Luisa Imberti, Alessandra Sottini, Eugenia Quiros-Roldan, Francesco Castelli, Camillo Rossi, Duilio Brugnoni, Andrea Biondi, Laura Rachele Bettini, Mariella D’Angio’, Paolo Bonfanti, Riccardo Castagnoli, Daniela Montagna, Amelia Licari, Gian Luigi Marseglia, Emily F. Gliniewicz, Elana Shaw, Dana E. Kahle, Andre T. Rastegar, Michael Stack, Katherine Myint-Hpu, Susan L. Levinson, Mark J. DiNubile, Daniel W. Chertow, Peter D. Burbelo, Jeffrey I. Cohen, Katherine R. Calvo, John S. Tsang, NIAID COVID-19 Consortium, Helen C. Su, John I. Gallin, Douglas B. Kuhns, Raphaela Goldbach-Mansky, Michail S. Lionakis, Luigi D. Notarangelo

**Affiliations:** 1 Laboratory of Clinical Immunology and Microbiology, National Institute of Allergy and Infectious Diseases (NIAID), National Institutes of Health (NIH), Bethesda, Maryland, USA.; 2 Biostatistics Research Branch, NIAID, NIH, Bethesda, Maryland, USA.; 3 Neutrophil Monitoring Laboratory, Leidos Biomedical Research, Inc, Frederick National Laboratory for Cancer Research, Frederick, Maryland, USA.; 4 Children’s Hospital of Pittsburgh, University of Pittsburgh Medical Center, Pittsburgh, Pennsylvania, USA.; 5 CREA Laboratory, Diagnostic Department, ASST Spedali Civili di Brescia, Brescia, Italy.; 6 Department of Infectious and Tropical Diseases, University of Brescia and ASST Spedali Civili di Brescia, Brescia, Italy.; 7 Direzione Sanitaria, ASST Spedali Civili di Brescia, Italy.; 8 Laboratorio Analisi Chimico-Cliniche, ASST Spedali Civili, Brescia, Italy.; 9 Pediatric Department and Centro Tettamanti-European Reference Network on Paediatric Cancer, European Reference Network on Haematological Diseases, and European Reference Network on Hereditary Metabolic Disorders-University of Milano-Bicocca-Fondazione MBBM, Monza, Italy.; 10 Department of Infectious Diseases, San Gerardo Hospital, University of Milano-Bicocca, Monza, Italy.; 11 Department of Pediatrics and; 12 Laboratory of Immunology and Transplantation, Fondazione IRCCS Policlinico San Matteo, Pavia, Italy.; 13 Department of Clinical, Surgical, Diagnostic and Pediatric Sciences, University of Pavia, Pavia, Italy.; 14 BioAegis Therapeutics, Inc, North Brunswick, New Jersey, USA.; 15 Critical Care Medicine Department, NIH Clinical Center, NIH, Bethesda, Maryland, USA.; 16 National Institute of Dental and Craniofacial Research, NIH, Bethesda, Maryland, USA.; 17 Laboratory of Infectious Diseases, NIAID, NIH, Bethesda, Maryland, USA.; 18 Hematology Section, Department of Laboratory Medicine, NIH Clinical Center, NIH, Bethesda, Maryland, USA.; 19 Laboratory of Immune System Biology and Clinical Genomics Program, NIAID, NIH, Bethesda, Maryland, USA.; 20 Center for Human Immunology, Autoimmunity, and Inflammation, NIAID, NIH, Bethesda, Maryland, USA.; 21 The NIAID COVID-19 Consortium is detailed in Supplemental Acknowledgments.

**Keywords:** COVID-19, Immunology, Chemokines, Cytokines

## Abstract

Immune and inflammatory responses to severe acute respiratory syndrome coronavirus 2 (SARS-CoV-2) contribute to disease severity of coronavirus disease 2019 (COVID-19). However, the utility of specific immune-based biomarkers to predict clinical outcome remains elusive. Here, we analyzed levels of 66 soluble biomarkers in 175 Italian patients with COVID-19 ranging from mild/moderate to critical severity and assessed type I IFN–, type II IFN–, and NF-κB–dependent whole-blood transcriptional signatures. A broad inflammatory signature was observed, implicating activation of various immune and nonhematopoietic cell subsets. Discordance between IFN-α2a protein and *IFNA2* transcript levels in blood suggests that type I IFNs during COVID-19 may be primarily produced by tissue-resident cells. Multivariable analysis of patients’ first samples revealed 12 biomarkers (CCL2, IL-15, soluble ST2 [sST2], NGAL, sTNFRSF1A, ferritin, IL-6, S100A9, MMP-9, IL-2, sVEGFR1, IL-10) that when increased were independently associated with mortality. Multivariate analyses of longitudinal biomarker trajectories identified 8 of the aforementioned biomarkers (IL-15, IL-2, NGAL, CCL2, MMP-9, sTNFRSF1A, sST2, IL-10) and 2 additional biomarkers (lactoferrin, CXCL9) that were substantially associated with mortality when increased, while IL-1α was associated with mortality when decreased. Among these, sST2, sTNFRSF1A, IL-10, and IL-15 were consistently higher throughout the hospitalization in patients who died versus those who recovered, suggesting that these biomarkers may provide an early warning of eventual disease outcome.

## Graphical abstract

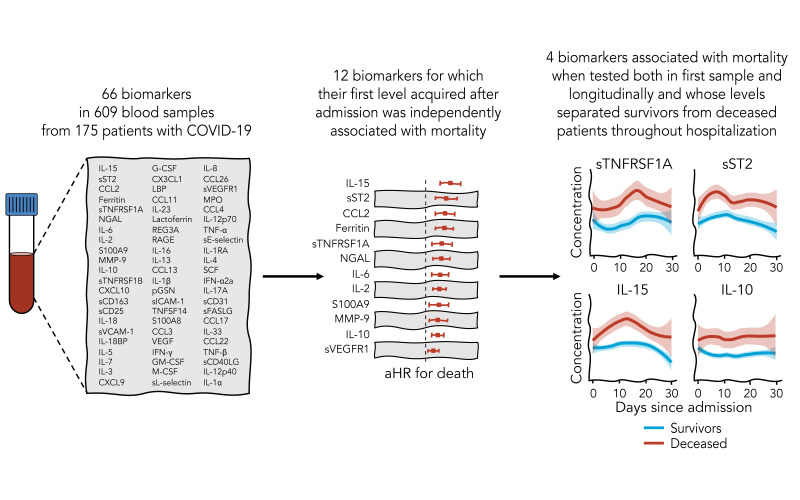

## Introduction

COVID-19 is a new human viral disease caused by SARS-CoV-2, an enveloped RNA beta coronavirus that emerged in China and spread globally, causing 1,460,000 deaths as of November 30, 2020 (1). The majority of affected individuals exhibit no or mild to moderate symptoms, but up to 15% of patients develop severe pneumonia with approximately 6% progressing to acute respiratory distress syndrome (ARDS) and multiorgan failure (2). Approximately one-fourth of subjects hospitalized with COVID-19–associated pneumonia require respiratory support in an intensive care unit (ICU), and the need for invasive mechanical ventilation (IMV) has been associated with high mortality (3–5). Older age, male sex, and the presence of certain comorbidities (e.g., diabetes, cardiovascular disease) have been identified as predictors of poor outcomes (5); however, even younger and generally healthy individuals can suffer from COVID-19–associated respiratory failure (6). Both virus-specific factors and host inflammatory responses have been implicated in determining disease severity and clinical outcome (7–9). It has been proposed that ineffective early innate antiviral response followed by impaired adaptive immune responses and hyperinflammation may lead to microthrombosis and tissue injury, resulting in ARDS, multiorgan failure, and death (10).

Abnormal blood levels of several pro- or antiinflammatory cytokines, chemokines, and other mediators have been associated with worse outcomes (4, 11–13). In particular, elevated IL-6 levels were shown to correlate with an increased risk of death (13, 14). Furthermore, patients requiring ICU admission exhibit higher plasma levels of IL-2, IL-7, IL-10, granulocyte colony-stimulating factor (G-CSF), CXCL10/IP-10, MCP-1/CCL2, MIP-1α/CCL3, and TNF-α (4, 14–19). Improved understanding of the immunopathogenesis of COVID-19 may allow for the identification of personalized prognostic markers and the design of personalized, targeted therapeutic interventions. However, most published studies to date have focused on a restricted panel of inflammatory mediators in relatively small patient cohorts with brief follow-up. In addition, limited information exists related to how levels of biomarkers may be affected by confounding factors, such as the presence and nature of comorbidities, various therapies (particularly immunomodulatory medications), and the timing of sampling relative to onset of infection and recovery/death.

We took a broad and methodologically multifaceted approach toward identifying immune-based biomarkers associated with clinical outcome by analyzing data on 66 soluble biomarkers in 175 patients hospitalized with COVID-19 of different degrees of severity. We explored the association between each biomarker and risk of mortality using 3 survival models of escalating scope. First, we modeled survival using the first biomarker measurement and adjusting for time from admission to sample collection. Second, we adjusted for potential confounders, including age, biomarker-modifying comorbidities, and receipt of immunomodulatory drugs. Third, we jointly modeled longitudinal patterns in biomarkers using all collected longitudinal samples and the association between survival and the expected value of each biomarker, again adjusting for potential confounders. We identified several biomarkers that were consistently associated with mortality across analyses after controlling for FDR. These data provide novel and important insights into the immunopathogenesis of severe COVID-19. If validated in larger independent cohorts, our findings may offer the opportunity to develop personalized strategies for risk assessment and individualized, tailored therapeutic intervention of hospitalized patients with severe COVID-19.

## Results

### Clinical characteristics of our cohort.

We enrolled 175 patients with confirmed COVID-19 (defined by SARS-CoV-2–positive PCR test from a respiratory swab and/or positive serology for SARS-CoV-2) who were hospitalized in 3 hospitals in the Lombardy region of Italy between February 25, 2020, and May 9, 2020. This period coincided with the early surge in COVID-19 cases in northern Italy that outstripped the capacity of available health care resources (20). The median age of patients was 60 years (IQR, 51–69 years) and 132 (75.4%) were male. Patients presented to the hospital after a median of 7 days from symptom onset (IQR, 5–10 days). The maximum severity of a patient’s COVID-19 infection during hospitalization was classified as previously described (21). A total of 122 patients (69.7%) had critical disease, while 23 (13.1%) had severe and 30 (17.1%) had mild or moderate disease. Information about comorbid medical conditions was available for 172 (98.3%) patients. At least 1 comorbidity was present in 140 patients (81.4%), with hypertension (37.2%) and diabetes (20.9%) being most common followed by malignancy and autoimmune disease (Supplemental Table 1; supplemental material available online with this article; https://doi.org/10.1172/jci.insight.144455DS1). During the course of hospitalization, 157 patients (89.7%) patients required some form of supplemental oxygen. Seventy patients (40%) were admitted to the ICU, nearly all of whom (*n* = 69) required IMV. The majority of patients (129, 73.7%) received varying combinations of azithromycin, hydroxychloroquine, and/or antiviral therapy (remdesivir, darunavir/ritonavir, or lopinavir/ritonavir). Detailed information on receipt of anticoagulation and immunomodulatory medications was available for 160 patients. Prophylactic or therapeutic anticoagulation was administered to 105 patients (65.6%) (Supplemental Table 1). Among immunomodulatory medications, corticosteroids were used in 93 patients (58.1%), the IL-6 receptor–targeted monoclonal antibody (mAb) tocilizumab in 40 (25%), the IL-1β–targeted mAb canakinumab in 7 (4.4%), and the IFN-γ–targeted mAb emapalumab in 1 (0.6%). Thrombotic complications during illness were documented in 22 patients (12.6%); deep venous thrombosis and/or pulmonary embolism accounted for nearly three-quarters of thrombotic complications, with the remaining cases consisting of stroke and myocardial infarction. Acute kidney injury developed in 41 patients (23.4%). As of July 15, 2020, 33 patients (18.9%) had died. Among these 33 patients, the median time from hospital admission to death was 25 days (IQR, 14–46 days). Among the 142 survivors, the median duration of hospitalization was 19 days (IQR, 12–35 days).

### Clinical factors and laboratory tests associated with differential mortality using univariable analysis in our cohort.

Among clinical factors, age greater than 65 years (HR, 3.96; 95% CI, 1.76–8.89), diabetes (HR, 2.54; 95% CI, 1.23–5.29), ICU admission (HR, 2.21; 95% CI, 1.01–4.85), and intubation (HR, 2.65; 95% CI, 1.17–5.99), but not sex, obesity, malignancy, or chronic liver or respiratory conditions, were associated with increased mortality (Supplemental Figure 1 and Supplemental Table 2). Among laboratory tests, elevated neutrophil-to-lymphocyte ratio, but not elevated levels of lactate dehydrogenase, C-reactive protein, or D-dimer, were associated with increased mortality. In contrast, decreased absolute lymphocyte counts were associated with increased mortality (Supplemental Figure 1 and Supplemental Table 2), consistent with previous reports (22, 23). Administration of anticoagulation was associated with reduced mortality (Supplemental Figure 1 and Supplemental Table 2). While corticosteroid use was not associated with a mortality benefit in the entirety of the patient cohort, improved survival was noted in the subset of intubated patients (Supplemental Figure 2), consistent with the findings of the RECOVERY trial (24).

### SARS-CoV-2 infection is associated with altered patterns in a wide range of immunologic effectors in blood that vary depending on disease severity.

To characterize the immunologic response in SARS-CoV-2 infection manifesting with different grades of severity, we measured the concentration of 66 biomarkers associated with monocyte/macrophage, inflammasome, NF-κB, and neutrophil activation; T cell activation and/or polarization; type I IFN and IFN response gene induction; endothelial integrity; and sepsis severity in the peripheral blood of COVID-19 patients and compared them with levels in healthy American volunteers (HVs). Cytokine and chemokine levels can vary considerably in their longitudinal trajectories during the course of COVID-19 as a function of the phase of the disease and receipt of immunomodulatory medications (15, 25). Therefore, we focused our initial analysis on 119 patients who underwent the first blood sampling within the initial 7 days of hospitalization (Supplemental Table 1 and Supplemental Figure 3).

### Monocyte/macrophage activation–associated biomarkers are markedly increased in COVID-19 patients.

**Figure 1:**
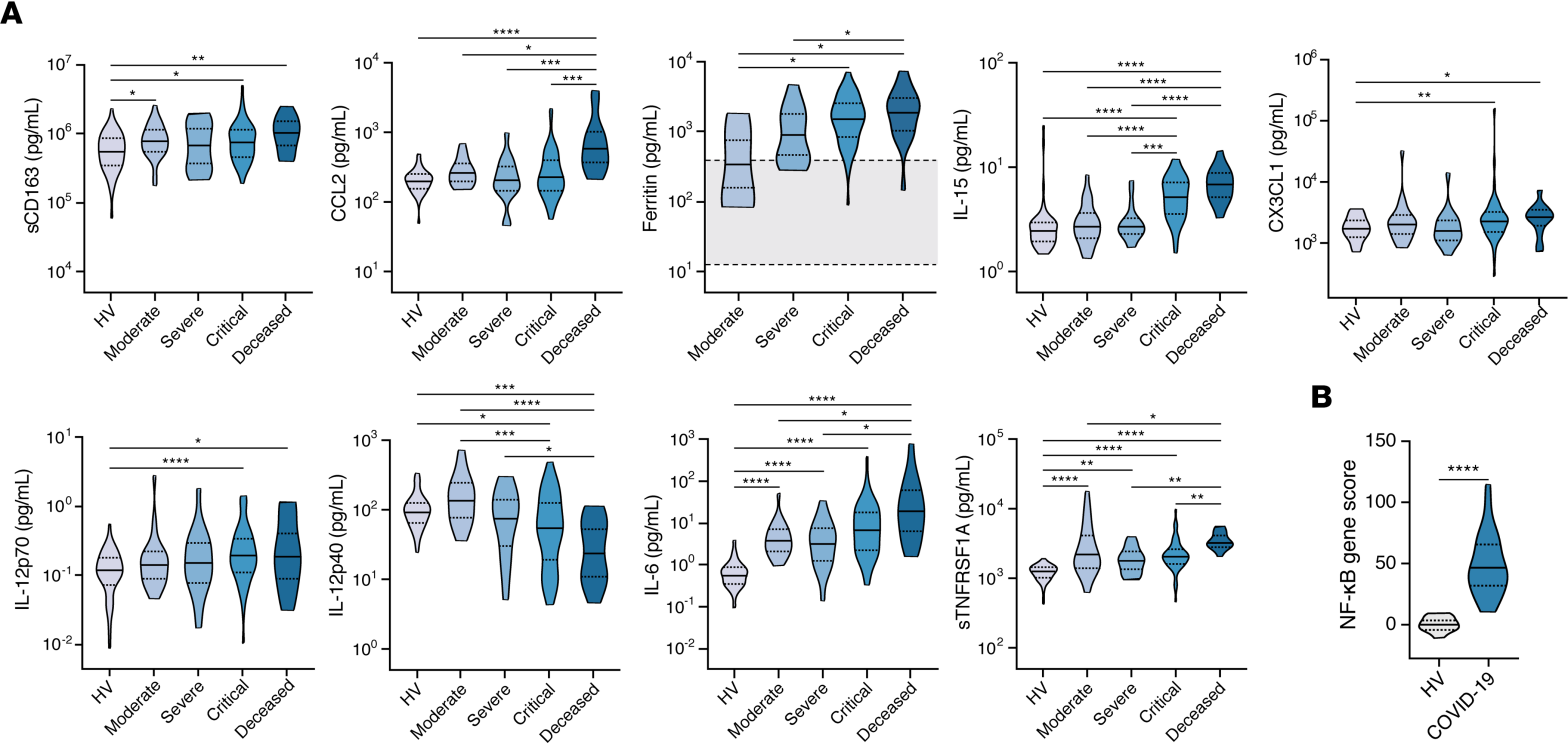
Biomarkers associated with activation of monocytes/macrophages and NF-κB signaling are markedly induced in COVID-19 patients. (**A**) Shown are levels of soluble CD163 (sCD163), CCL2, ferritin, IL-15, CX3CL1, IL-12p70, IL-12p40, IL-6, and sTNFRSF1A in peripheral blood of COVID-19 patients with various severity groups (*n* = 94–119 depending on the biomarker) relative to healthy volunteers (HV; *n* = 45–60 depending on the biomarker). Ferritin concentrations were determined by clinical assays performed in Italian hospitals. The area shaded in gray reflects the normal range for HVs reported by the clinical laboratory. Groups were compared by Kruskal-Wallis test. When *P* < 0.05, pairwise comparisons were made using Dunn’s test with Benjamini-Hochberg adjustment for multiple comparisons. (**B**) Expression of 11 NF-κB–regulated genes was measured by NanoString and expressed as summary *z* scores in whole blood of COVID-19 patients (*n* = 29) and HVs (*n* = 22). Groups were compared by an unpaired Student’s *t* test. *P < 0.05, **P < 0.01, ***P < 0.001, ****P < 0.0001.

Biomarkers associated with monocyte/macrophage activation were mostly upregulated in the blood of COVID-19 patients (Figure 1A, Supplemental Figure 4, and Supplemental Table 3), consistent with prior reports (14, 15). Comparing the concentration of biomarkers across severity groups and relative to HVs, several distinct patterns emerged. For example, the concentrations of MIP-1α/CCL3, soluble CD163 (sCD163), and M-CSF were elevated in all COVID-19 patients compared with HVs, regardless of severity. In contrast, MCP-1/CCL2 and MIP-1β/CCL4 were elevated selectively in patients who eventually succumbed to COVID-19 but not in those with milder illness. Ferritin, IL-15, CX3CL1 (also known as fractalkine), and IL-12p70 were elevated in patients with critical disease who survived as well as those who died. Notably, IL-12p40 levels were inversely correlated with disease severity. Consistent with the observed increased levels of monocyte activation–associated biomarkers, and in agreement with recent reports (26, 27), peripheral blood monocytes of COVID-19 patients exhibited extensive vacuolization (Supplemental Figure 5A), and the MFIs of CD169, and of CD63, CD11b, and b558, were greater in peripheral blood CD14^+^ monocytes of COVID-19 patients (*n* = 2) relative to HVs (Supplemental Figure 6).

Excessive inflammasome activation–mediated IL-1β and IL-18 production is also reflective of monocyte/macrophage activation and has been associated with cytokine release syndromes, viral and bacterial sepsis, and autoinflammatory conditions (28–30). The IL-1 receptor antagonist anakinra reduces mortality in patients with sepsis who show signs of macrophage activation syndrome (MAS) (31) and has shown promise in nonrandomized studies of patients with COVID-19 who suffer from hypoxemia or secondary hemophagocytic lymphohistiocytosis (32, 33). Some, but not all, prior studies found increased IL-1β levels in COVID-19 patients compared with HVs, but most studies showed little or no correlation between IL-1β levels and severity of COVID-19 (14, 15, 17). In our cohort, levels of IL-1β and IL-18 were higher in patients with COVID-19 compared with HVs. IL-1 receptor antagonist (IL-1RA) and IL-18 binding protein (IL-18BP), both negative regulators of IL-1β and IL-18 signaling, respectively, were also upregulated (Supplemental Figure 7A and Supplemental Table 3). Of note, the levels of IL-1β in COVID-19 patients were comparable to those seen in monogenic autoinflammatory disorders of IL-1β excess, whereas the levels of IL-18 were lower in COVID-19 patients relative to the levels observed in autoinflammatory IL-18opathies that predispose to MAS (Supplemental Figure 7B). However, we found no difference in the levels of IL-1β, IL-1RA, IL-18, and IL-18BP among COVID-19 severity groups (Supplemental Figure 7A). Taken together, these results indicate that SARS-CoV-2 infection results in significant increases in biomarkers associated with monocyte/macrophage activation and inflammasome induction, irrespective of disease severity.

### NF-κB–dependent biomarkers including IL-6 and TNF superfamily mediators are increased in COVID-19 patients.

In addition to the chemokines MCP-1/CCL2 and MIP-1α/CCL3, which were increased in COVID-19 patients (Figure 1A), IL-6 and TNF-α production also depends on NF-κB activation. IL-6, in particular, has received considerable attention as a potential biomarker of COVID-19 severity and mortality (15, 19, 25, 34–36). In line with prior reports (15), IL-6 was ~1 log higher in COVID-19 patients compared with HVs, with the highest levels noted in the most severely ill patients (Figure 1A and Supplemental Table 3). Consistent with some, but not all, prior studies (10, 14–16, 37, 38), TNF-α levels were increased in COVID-19 patients compared with HVs, but levels did not differ among severity groups (Figure 1); in contrast, TNF-β levels were comparable between HVs and COVID-19 patients (Supplemental Figure 4 and Supplemental Table 3). Of note, levels of TNFSF14 (also known as LIGHT) were higher in COVID-19 patients with severe compared with those with moderate disease, consistent with a recent report (39). Levels of the soluble TNF receptors, sTNFRSF1A and sTNFRSF1B, increased in a stepwise fashion with greater severity, and their highest concentrations were observed in patients who eventually succumbed to COVID-19 (Figure 1A). In keeping with the observed increase in biomarkers associated with NF-κB activation, a transcriptional score derived from the analysis of 11 NF-κB–regulated genes was significantly upregulated in whole blood of COVID-19 patients relative to HVs (Figure 1B).

### Neutrophil activation–associated biomarkers are enriched in COVID-19 patients with more severe disease.

**Figure 2:**
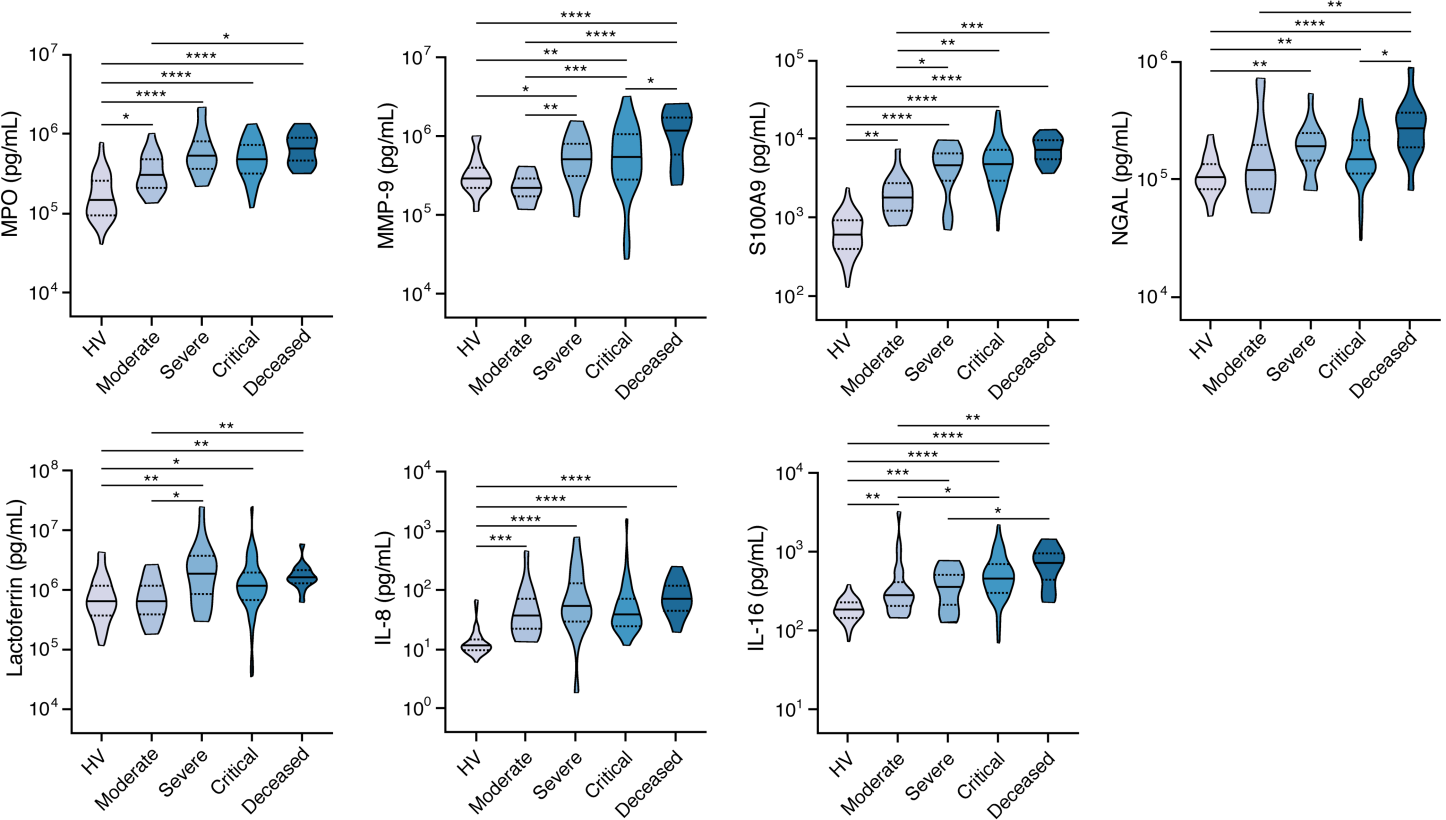
Neutrophil activation–associated biomarkers are increased in COVID-19 patients with more severe disease. Shown are levels of MPO, MMP-9, S100A9, NGAL, lactoferrin, IL-8, and IL-16 in peripheral blood of COVID-19 patients with various severity groups (*n* = 80–119 depending on the biomarker) relative to healthy volunteers (HV; *n* = 12–60 depending on the biomarker). Groups were compared by Kruskal-Wallis test. When *P* < 0.05, pairwise comparisons were made using Dunn’s test with Benjamini-Hochberg adjustment for multiple comparisons. *P < 0.05, **P < 0.01, ***P < 0.001, ****P < 0.0001.

Activated neutrophils, including formation of neutrophil extracellular traps, have been implicated in the immunopathogenesis of severe COVID-19 (40–43). To investigate the association between a variety of neutrophil activation–associated biomarkers and COVID-19 severity, we measured the concentrations of myeloperoxidase (MPO), MMP-9, S100 calcium binding protein A9 (S100A9), lipocalin-2 (also known as neutrophil gelatinase-associated lipocalin, NGAL), lactoferrin, IL-8, and IL-16 in SARS-CoV-2–infected patients and HVs (Figure 2 and Supplemental Table 3). All neutrophil biomarkers were higher in COVID-19 patients compared with HVs. For MPO, S100A9, IL-8, and IL-16, this difference was evident even when comparing HVs and patients with moderate disease severity. For all neutrophil markers except IL-8, levels further increased as COVID-19 severity increased, and a progressive, stepwise increase was observed for S100A9 and IL-16. In keeping with increased levels of biomarkers associated with neutrophil activation and increased IL-8 levels, the MFI of the granule protein CD66b was greater and the MFI of the IL-8 receptor CXCR2 was lower in peripheral blood neutrophils of COVID-19 patients (*n* = 2) relative to HVs (Supplemental Figure 8), and peripheral blood neutrophils of COVID-19 patients exhibited pronounced vacuolization (Supplemental Figure 5B).

Because neutrophilia is common in COVID-19 patients (11), we evaluated levels of the colony-stimulating growth factors G-CSF, GM-CSF, and stem cell factor (SCF) and found different patterns depending on disease severity. Specifically, while GM-CSF was increased in COVID-19 patients across all severity strata relative to HVs, G-CSF was increased only in patients with critical disease; SCF was increased only in patients with moderate disease (Supplemental Figure 9 and Supplemental Table 3).

### Th1 more than Th2 immune response–associated biomarkers are increased in patients with COVID-19, while soluble FAS ligand and soluble CD40 ligand are decreased.

**Figure 3:**
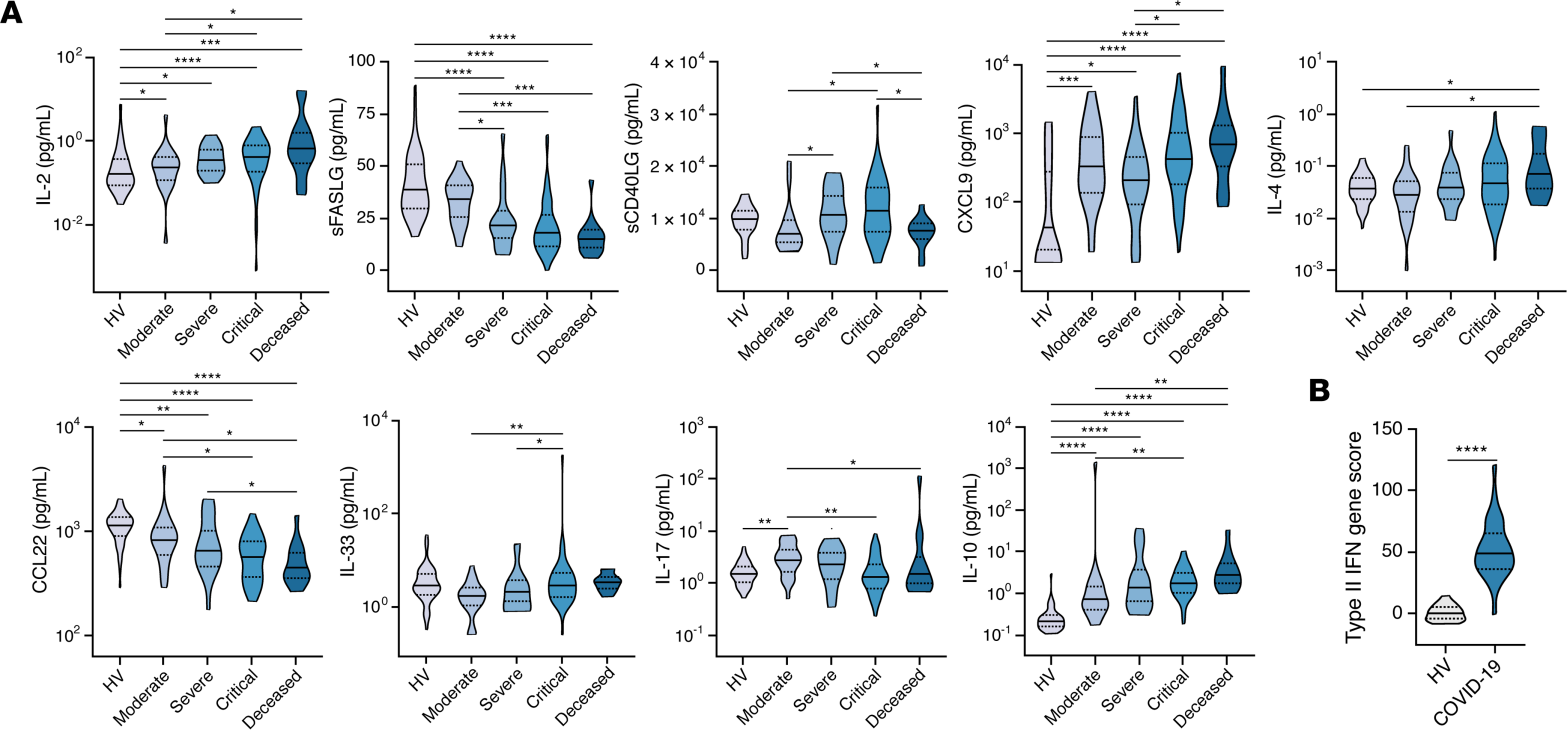
Th1-type immune response–associated biomarkers are predominantly increased in patients with COVID-19 relative to Th2 and Th17 immune response–associated biomarkers, while sFASLG and sCD40LG are decreased. (**A**) Shown are levels of IL-2, sFASLG, sCD40LG, CXCL9, IL-4, CCL22, IL-33, IL-17, and IL-10 in peripheral blood of COVID-19 patients with various severity groups (*n* = 94–119 depending on the biomarker) relative to healthy volunteers (HV; *n* = 34–60 depending on the biomarker). Groups were compared by Kruskal-Wallis test. When *P* < 0.05, pairwise comparisons were made using Dunn’s test with Benjamini-Hochberg adjustment for multiple comparisons. (**B**) Expression of 15 type II IFN–regulated (IFN-γ–regulated genes was measured by NanoString and expressed as summary *z* scores in whole blood of COVID-19 patients (*n* = 29) and HVs (*n* = 22). Groups were compared by an unpaired Student’s *t* test. *P < 0.05, **P < 0.01, ***P < 0.001, ****P < 0.0001.

To interrogate the potential role of T cell activation–related biomarkers in COVID-19 immunopathogenesis, we measured levels of IL-2, sCD25 (or sIL-2Rα), soluble CD40 ligand (sCD40LG), soluble FAS ligand (sFASLG), IL-7, and IL-3 (Figure 3A, Supplemental Figure 10, and Supplemental Table 3). IL-2 and sCD25 levels were increased in COVID-19 patients compared with HVs with minor or no significant differences between severity groups. In contrast, a small albeit statistically significant increase in IL-7 levels was noted in patients with critical COVID-19 (Supplemental Table 3). Notably, as observed with IL-12p40 (Figure 1A), sFASLG and sCD40LG were significantly reduced in patients who died of COVID-19. In fact, for sFASLG, the levels decreased in a stepwise manner with progression from moderate to critical infection. No differences in IL-3 levels were observed between COVID-19 patients and HVs.

Th1-type immune responses are critical for host defense against viruses and other intracellular pathogens but, when in excess, can also instigate hyperinflammation and tissue injury. Of note, IFN-γ and the IFN-γ–inducible chemokine CXCL9 were significantly increased in COVID-19 patients compared with HVs across severity groups. In agreement, a transcriptional score derived from the analysis of 15 IFN-γ–regulated genes was significantly upregulated in whole blood of COVID-19 patients relative to HVs (Figure 3B and Supplemental Table 3). Flow cytometric analysis of unstimulated whole-blood samples revealed a significant increase in the percentage of IFN-γ^+^CD8^+^ T cells in COVID-19 patients relative to HVs, while IFN-γ production was comparable in CD4^+^ T cells, NK cells, and NKT cells between COVID-19 patients and HVs (Supplemental Figure 11A).

We next examined Th2-type immunity–associated biomarkers (Figure 3A, Supplemental Figure 10, and Supplemental Table 3) because excessive type 2 responses may promote immunopathology during severe respiratory viral infections as previously shown for respiratory syncytial virus bronchiolitis (44). The levels of IL-4, IL-13, CCL11 (also known as eotaxin-1), and MCP-4/CCL13 were comparable between HVs and COVID-19 patients with mild/moderate and severe disease but were increased in the subset of COVID-19 patients who died, consistent with a previous report showing an upward trend for IL-4 and IL-13 over the course of the disease in patients with severe COVID-19 (15). Levels of CCL26 (also known as eotaxin-3) were increased in COVID-19 patients and correlated with disease severity. In contrast, levels of CCL17 (also known as TARC) and CCL22 (also known as MDC) were reduced in COVID-19 patients relative to HVs, with an inverse association observed between CCL22, but not CCL17, levels and COVID-19 severity. Levels of IL-5 were not significantly influenced by SARS-CoV-2 infection, whereas IL-33 was increased in patients with severe and critical COVID-19 but not in patients who died from COVID-19.

We found modest increases in the Th17-type immune response–associated biomarkers IL-17 and IL-23 in COVID-19 patients and a more significant increase in IL-10 levels in all severity groups, with the highest concentrations observed in patients who succumbed to COVID-19 (Figure 3A, Supplemental Figure 10, and Supplemental Table 3), as previously reported (15, 19, 45). Of note, flow cytometric analysis of unstimulated whole-blood samples revealed no significant enrichment in the percentage of IL-4^+^ or IL-17A^+^ lymphoid cell populations in COVID-19 patients relative to HVs (Supplemental Figure 11B). Collectively, these data show that Th1-type immune response–associated biomarkers are predominantly increased over Th2 and Th17 immune response–associated biomarkers in COVID-19 patients.

### Biomarkers associated with endothelial integrity and sepsis severity are increased in COVID-19, while plasma gelsolin and IL-1α levels are decreased in patients who die from COVID-19.

**Figure 4:**
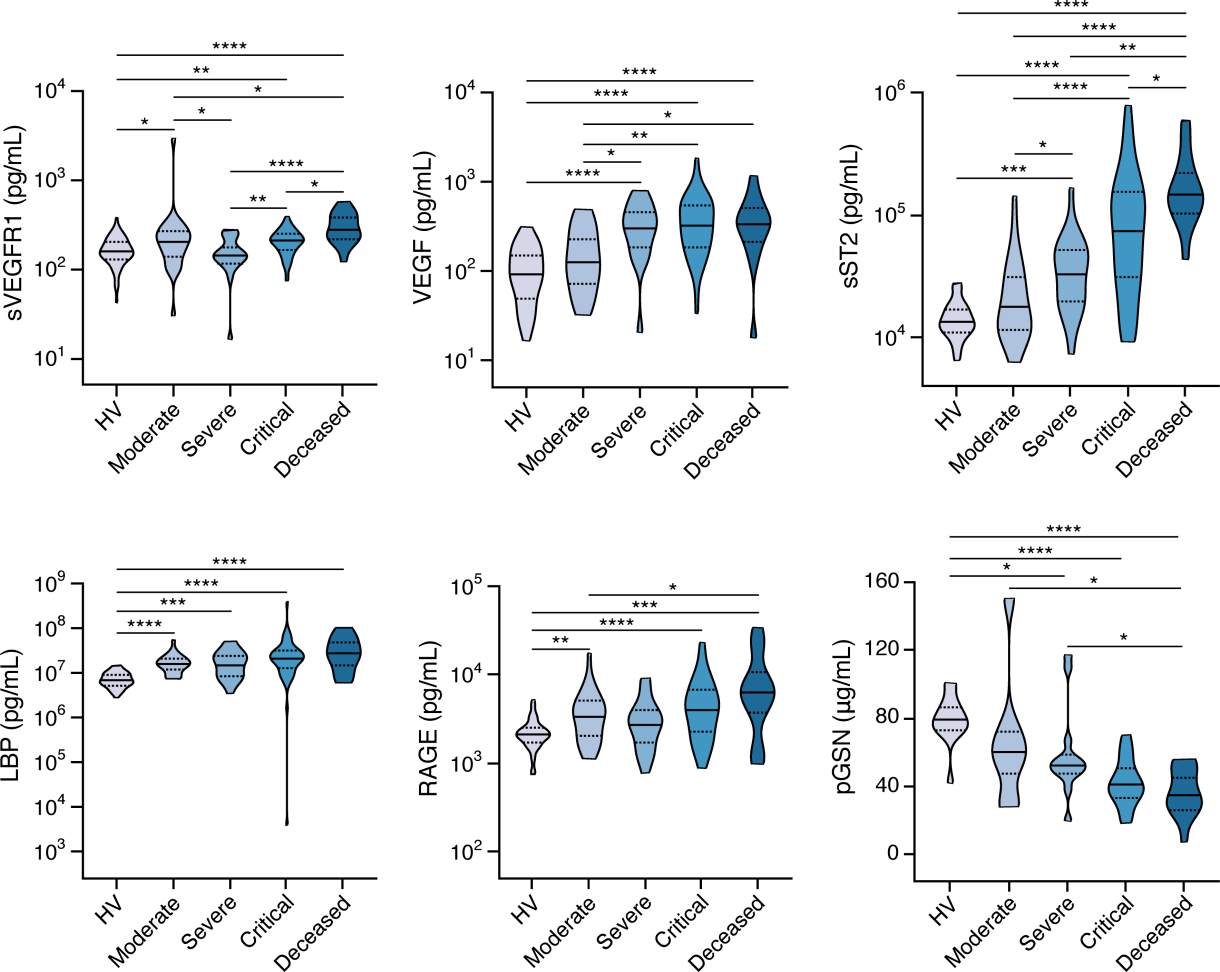
Abnormal levels of biomarkers associated with endothelial integrity and sepsis severity in COVID-19 patients. Shown are levels of soluble VEGF receptor 1 (sVEGFR1), VEGF, sST2 LPS binding protein (LBP), receptor of advanced glycation end products (RAGE), and plasma gelsolin (pGSN) in peripheral blood of COVID-19 patients with various severity groups (*n* = 93–119) relative to healthy volunteers (HV; *n* = 14–60 depending on the biomarker). Groups were compared by Kruskal-Wallis test. When *P* < 0.05, pairwise comparisons were made using Dunn’s test with Benjamini-Hochberg adjustment for multiple comparisons. *P < 0.05, **P < 0.01, ***P < 0.001, ****P < 0.0001.

Leukocyte migration from the bloodstream to infected tissues requires their highly coordinated interaction with the endothelial surface (46). Endothelial dysfunction is known to underlie several complications of infection (including thrombosis), which have been frequently observed in patients with COVID-19 (47). Analysis of several biomarkers of endothelial function revealed increased blood levels of soluble VCAM-1 (sVCAM-1) and vascular endothelial growth factor (VEGF) and decreased levels of soluble L selectin shed from activated neutrophils (sL selectin; also known as sCD62L) and soluble CD31 (sCD31; also known as sPECAM-1) in patients with COVID-19 compared with HVs (Figure 4, Supplemental Figure 12, and Supplemental Table 3). A statistically significant increase in sVEGFR1 levels above HVs was apparent in the most severely ill patients with COVID-19, while modest changes were observed for soluble ICAM-1 (sICAM-1) and soluble E selectin (sE selectin; also known as sCD62E) (Figure 4, Supplemental Figure 12, and Supplemental Table 3).

S100A9; LBP; soluble ST2 (sST2), which serves as a decoy receptor for IL-33; and RAGE (also known as AGER) have been used as prognostic biomarkers in sepsis (48–53). sST2 levels were increased in COVID-19 patients and were strongly correlated with disease severity, with marked elevations seen in patients who died (Figure 4 and Supplemental Table 3). RAGE, a biomarker of tissue damage that interacts with S100A9 (54), was also elevated in COVID-19 patients, especially in those who died (Figure 4 and Supplemental Table 3), as also seen for S100A9 (Figure 2). In contrast, LBP was increased similarly in COVID-19 patients across all severity groups. Notably, pGSN, a biomarker thought to curtail inflammation and platelet aggregation–associated coagulation whose low levels have been seen in patients with sepsis and have been associated with impaired lung function and death (55), was significantly decreased with increasing disease severity, with the lowest pGSN levels seen in patients who subsequently died (Figure 4 and Supplemental Table 3).

We also measured the epithelial cell–derived biomarkers S100A8, regenerating islet-derived protein 3 alpha (REG3A), and IL-1α (Supplemental Figure 13 and Supplemental Table 3) and found both S100A8 and REG3A levels to be modestly increased in COVID-19 patients relative to HVs across all severity categories. IL-1α levels were increased in patients with moderate and severe disease but significantly decreased in patients who died. Taken together, these data indicate that endothelial cell– and sepsis-associated biomarkers are increased during COVID-19.

### Type I IFN induction is seen across all COVID-19 severity groups, but the transcriptional response of type I IFN genes in circulating cells is disproportionally low.

Type I IFN signaling is critical for mounting effective antiviral immune responses. Indeed, monogenic disorders in the type I IFN signaling pathway or autoantibodies against type I IFNs have been associated with development of severe viral infections (56–64). However, excessive type I IFN signaling leads to chronic inflammation, as highlighted by several monogenic autoinflammatory disorders (65), and may contribute to immunopathology during the late phases of SARS-CoV-1 and SARS-CoV-2 infections (66). It has been hypothesized that a biphasic type I IFN response consisting of an early protective response and a subsequent immunopathogenic response may operate during COVID-19, and randomized clinical trials are currently underway to evaluate the efficacy of either IFN-β or JAK inhibitors, which inhibit type I IFN signaling, in COVID-19.

**Figure 5:**
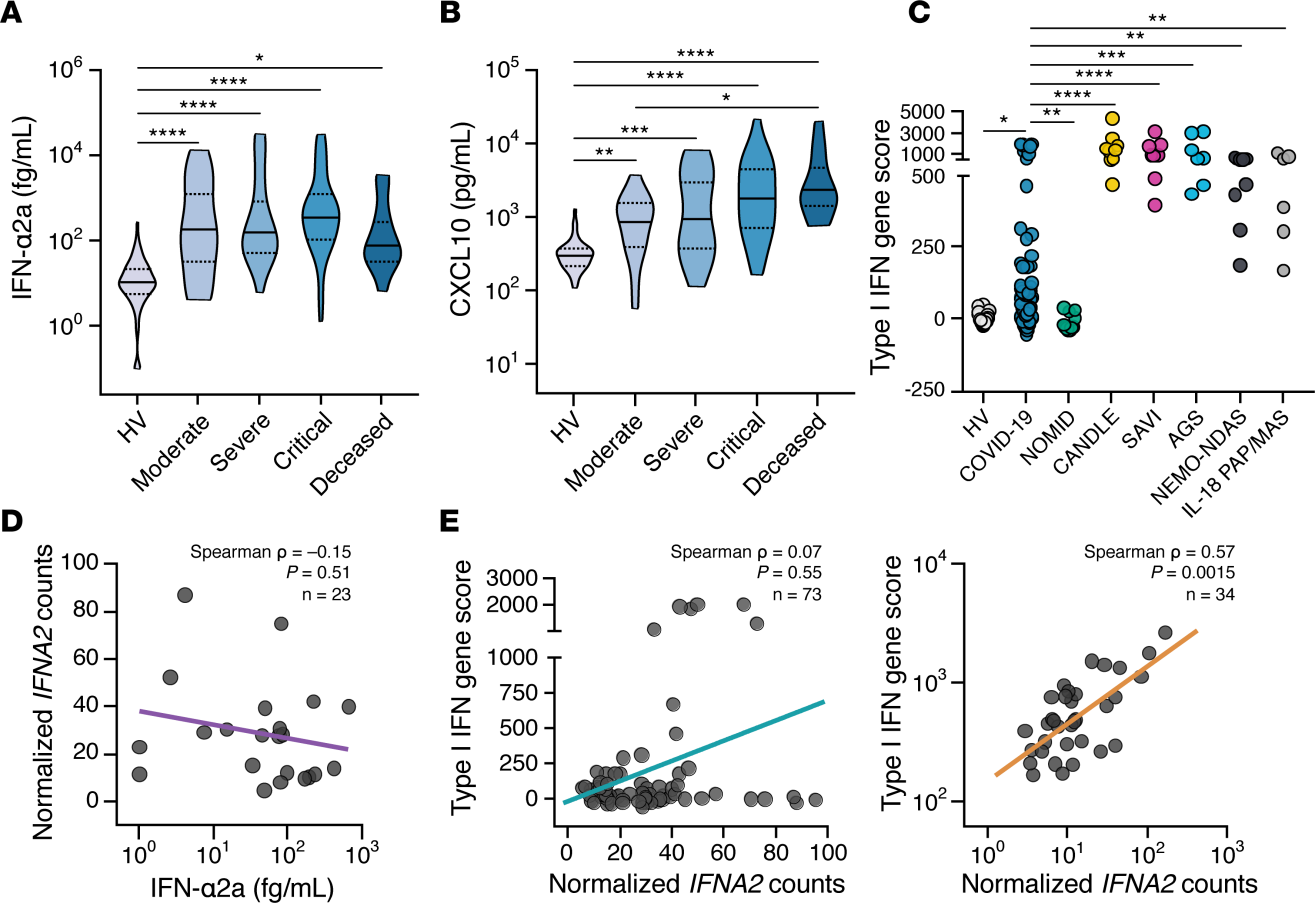
Type I IFN mediators are increased in COVID-19 patients, but the transcriptional response of type I IFN genes in circulating immune cells is disproportionally low. (**A**–**B**) Shown are (**A**) IFN-α2a and (**B**) CXCL10 levels in peripheral blood of COVID-19 patients with various severity groups (*n* = 94–114 depending on the biomarker) relative to healthy volunteers (HV; *n* = 45–67 depending on the biomarker). Groups were compared by Kruskal-Wallis test. When *P* < 0.05, pairwise comparisons were made using Dunn’s test with Benjamini-Hochberg adjustment for multiple comparisons. *P < 0.05, **P < 0.01, ***P < 0.001, ****P < 0.0001. (**C**) Expression of 28 type I IFN–induced genes was measured by NanoString and expressed as log_10_-transformed summary z scores. Shown is comparison of HVs (*n* = 22), COVID-19 patients (*n* = 84), and patients with the NLRP3 inflammasomopathy NOMID (*n* = 11); and the type I IFNopathies CANDLE (*n* = 9), SAVI (*n* = 9), and AGS (*n* = 7); the CANDLE mimic NEMO-NDAS (*n* = 9); and the IL-18opathy IL-18 PAP/MAS (*n* = 6). NOMID, neonatal onset multisystem inflammatory disease; CANDLE, chronic atypical neutrophilic dermatosis with lipodystrophy and elevated temperature; SAVI, STING-associated vasculopathy with onset in infancy; AGS, Aicardi-Goutières syndrome; NEMO-NDAS, NF-κB essential modulator-deleted exon 5 autoinflammatory syndrome; IL18 PAP/MAS, IL-18–mediated pulmonary alveolar proteinosis and macrophage activation syndrome. (**D**) Correlation of the transcript levels of *IFNA2* in whole blood with blood levels of IFN-α2a in patients with COVID-19 (*n* = 22). (**E**) Correlation of the 28 type I IFN–induced gene score with transcript levels of *IFNA2* in patients with COVID-19 (left panel) (*n* = 73) compared with the indicated type I IFNopathies (right panel) (*n* = 34).

Levels of IFN-α2a and the IFN-inducible chemokine CXCL10/IP-10 were significantly induced in the blood of COVID-19 patients across all severity groups (Figure 5, A and B, and Supplemental Table 3). While no differences were noted in IFN-α2a levels among patients of different severity groups, CXCL10 levels were greater in patients who succumbed to COVID-19 relative to those with moderate disease. We next examined a transcriptional score derived from the analysis of 28 type I IFN–regulated genes, which we have previously used to characterize monogenic type I IFNopathies (67). Notably, although a subset of COVID-19 patients had increased type I IFN scores relative to HVs, we found that the type I IFN score of COVID-19 patients was significantly lower than that observed in monogenic type I IFNopathies (Figure 5C). In addition, we found the normalized transcriptional levels of *IFNA2* of circulating leukocytes to be uncorrelated with the IFN-α2a blood levels (Figure 5D). In addition, normalized *IFNA2* transcripts only weakly correlated with the 28-gene type I IFN score in COVID-19 patients (Spearman’s = 0.07; *P* = 0.55), in contrast to their significant correlation in patients with monogenic IFNopathies (Spearman’s *P* = 0.57; *P* = 0.0015) (Figure 5E). The low transcriptional levels of *IFNA2* detected in blood are consistent with decreased numbers of circulating plasmacytoid DCs (pDCs) and impaired production of type I IFN by circulating pDCs in COVID-19 patients reported by others (10, 16, 19).

### Immunologic effectors as biomarkers associated with mortality in univariable analysis.

As mentioned earlier (Supplemental Figure 1) and previously described (2, 23), several clinical risk factors and laboratory tests have demonstrated utility in identifying patients at risk of death following SARS-CoV-2 infection. Having characterized the patterns of levels of 66 immunologic effectors in COVID-19 patients with different disease severities, we next asked which of these biomarkers might exhibit potential utility in identifying patients at risk for death after SARS-CoV-2 infection.

Using patients’ first sample from the 119 patients who had the first available sample collected within 7 days of hospitalization, we performed univariable analysis for each of the 66 biomarkers to identify which of them correlated with mortality. We found 12 biomarkers for which increased levels in the initial sample were associated with increased mortality (Supplemental Figure 14 and Supplemental Table 4). These biomarkers included 3 neutrophil activation–associated biomarkers (MMP-9, NGAL, and S100A9), 3 Th2-associated biomarkers (CCL26, CCL13, and CCL11), 3 monocyte/macrophage activation– and/or NF-κB activation–associated biomarkers (MCP-1/CCL2, sTNFRSF1A, IL-6), as well as sST2, IL-2, and sVEGFR1.

### A subset of immunologic biomarkers correlates with mortality in multivariable analysis.

**Figure 6:**
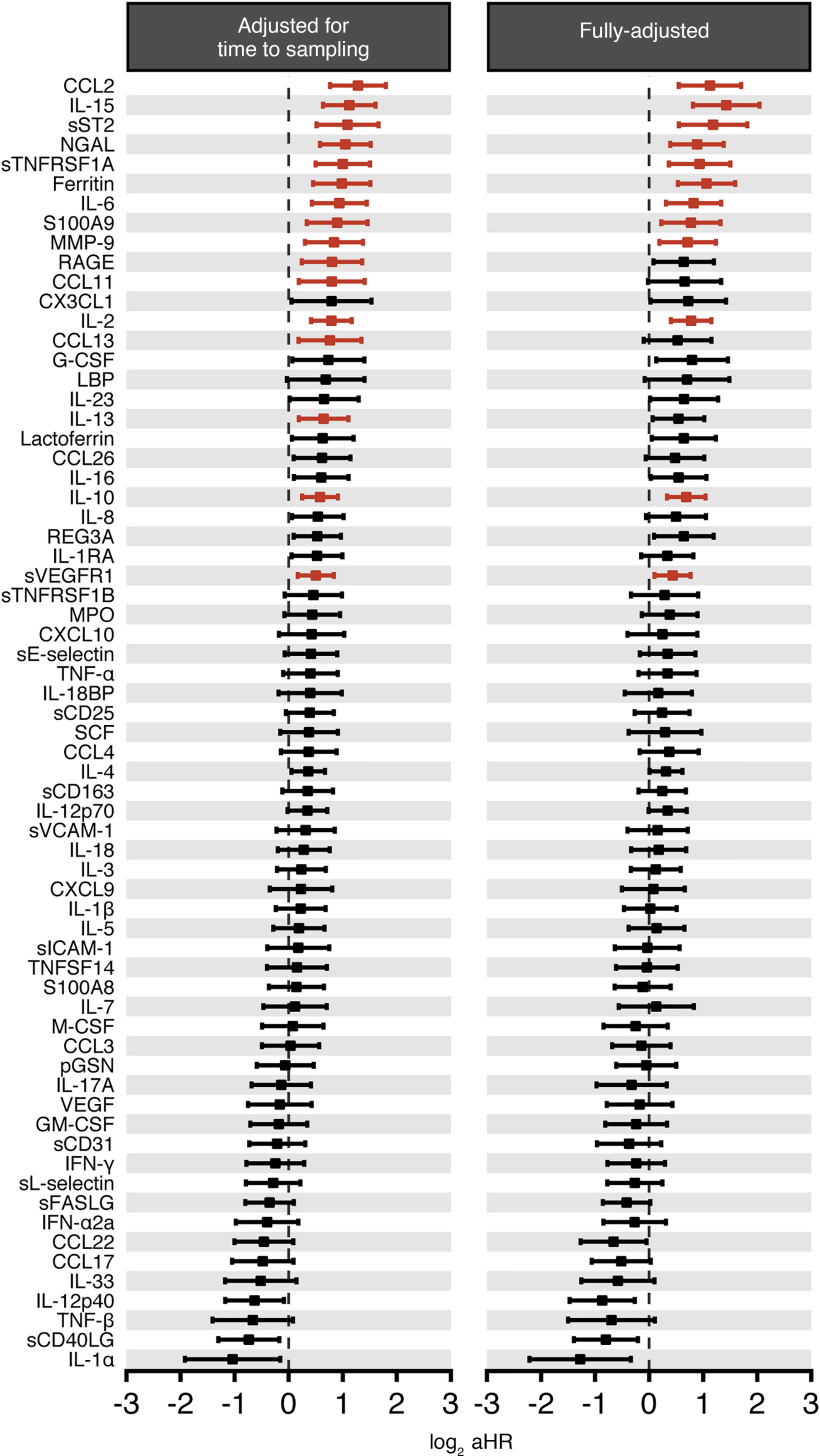
A subset of immune-based biomarkers is associated with mortality in COVID-19 patients in multivariable analyses. Shown are forest plots and adjusted HRs (aHRs) of all 66 tested biomarkers and their association with mortality during COVID-19 by multivariable analysis, irrespective of when the first sample was collected relative to the hospital admission when adjusting for (left panel) the time of sample collection relative to hospital admission or (right panel) the time of sample collection relative to hospital admission with age, chronic kidney disease, and receipt of immunomodulatory medications. For biomarkers significantly associated with mortality (i.e., *q* < 0.025), aHR CIs are shown in red.

We next expanded the analysis to include all 175 patients irrespective of whether the first available sample for analysis was collected within the initial 7 days of their hospitalization, adjusting for the time of sample collection relative to admission (Supplemental Table 4). The same 12 biomarkers were associated with mortality as previously shown in the univariable model that evaluated the 119 patients who had the first available sample collected within 7 days of hospitalization (Figure 6, left panel; Supplemental Figure 14; and Supplemental Table 4), with MCP-1/CCL2 emerging as the biomarker with the highest aHR (aHR, 2.43; 95% CI, 1.7–3.48) among all 66 biomarkers. In this analysis, we found 4 additional biomarkers whose increased levels in the initial sample were associated with increased mortality: IL-15, ferritin, RAGE, and IL-13. The following commonly measured biomarkers did not have a significant association with risk of death in this analysis: TNF-α, IFN-γ, IL-1β, IL-4, IL-8, and IL-18 (Supplemental Table 4).

Certain clinical factors may affect the levels of immunologic effectors and, as a result, confound their observed association with mortality without adjustment. Accordingly, we sought to identify potential clinical factors that could confound the association between biomarkers and survival for inclusion in our multivariable model. We fit unadjusted models estimating the associations between 20 clinical factors, including age, sex, and various comorbidities, and the levels of the 66 measured biomarkers (Supplemental Table 5). Age and chronic kidney disease were associated with the vast majority of alterations observed in biomarker levels (approximately 65%) and hence were included as covariates in our adjusted models. Additionally, receipt of immunomodulatory treatment with corticosteroids and/or tocilizumab, and/or canakinumab, was also included as a covariate because these drugs are known to potentially affect the levels of immunologic effectors (68–71).

In this multivariable analysis, we found 12 biomarkers for which increased levels were associated with increased mortality (Figure 6, right panel, and Supplemental Table 4). These biomarkers included 5 monocyte/macrophage activation–associated and/or NF-κB activation–associated biomarkers, including IL-15, which exhibited the highest aHR (2.66; 95% CI, 1.74–4.06) among all 66 biomarkers, and MCP-1/CCL2, sTNFRSF1A, ferritin, and IL-6, 3 neutrophil activation–associated biomarkers (NGAL, S100A9, and MMP-9), 2 T cell–associated biomarkers (IL-2 and IL-10), as well as sST2 and sVEGFR1. Interestingly, TNF-α, IFN-γ, and IL-1β levels remained nonstatistically associated with death in the multivariable analysis that included the entire patient cohort after adjusting for age, chronic kidney disease, and receipt of immunomodulatory medications (Supplemental Table 4).

### Longitudinal biomarker analysis and association with mortality.

To assess mortality while controlling for the longitudinal trajectory of the biomarkers over time, we employed a shared parameter joint model to describe trends in each biomarker over time and the association between the biomarker and a patient’s risk of death. These models combine a longitudinal mixed effects model fit to repeated measurements of a biomarker with a survival model estimating time to death (72, 73). Joint modeling in this analysis was conducted under a Bayesian framework.

**Figure 7:**
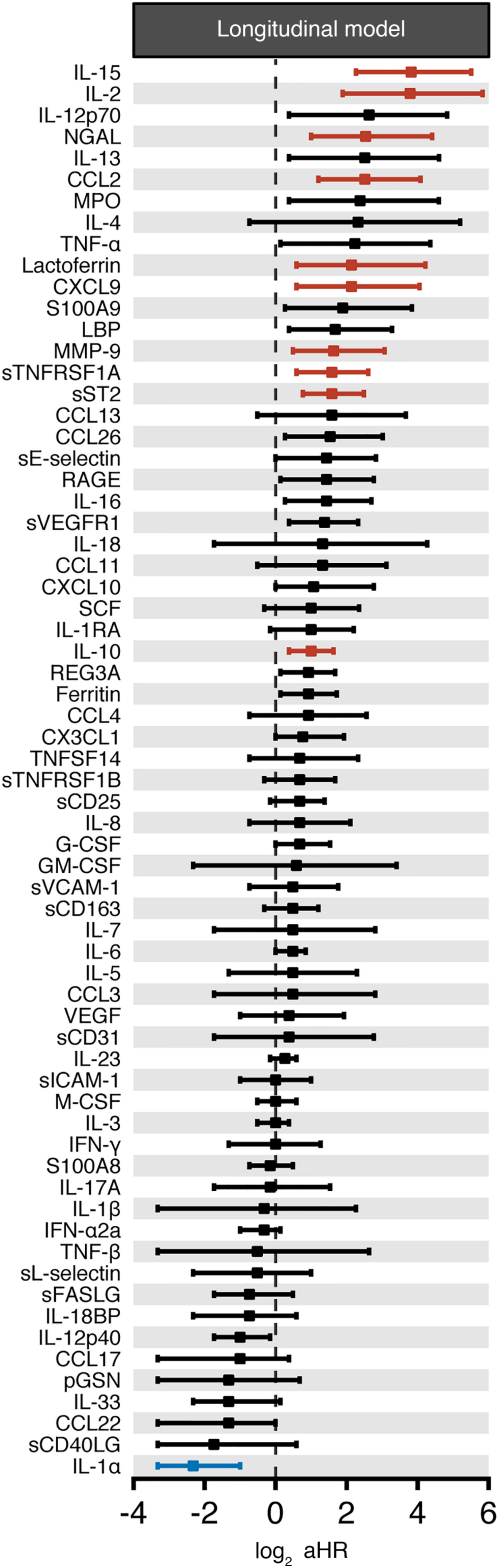
Association between the longitudinal trajectory of biomarkers and the risk of death after COVID-19. Shown are forest plots of the immune-based biomarkers (*n* = 66) whose longitudinal trajectories were significantly associated with increased patient mortality after controlling the FDR irrespective of when the first sample was collected relative to the hospital admission. aHR CIs for biomarkers significantly associated with mortality (i.e., *q* < 0.025) are shown in red when aHR > 1 and in blue when aHR < 1. aHR CIs for biomarkers with *q* > 0.025 are shown in black.

We found 11 biomarkers had a statistically significant association with increased patient mortality after controlling the FDR (Figure 7). Ten biomarkers were associated with increased mortality, and 1 was associated with decreased mortality (Supplemental Table 6). The 10 biomarkers significantly associated with increased mortality in this longitudinal analysis included 3 monocyte/macrophage activation–associated biomarkers (IL-15, MCP-1/CCL2, and sTNFRSF1A), 3 neutrophil activation–associated biomarkers (NGAL, MMP-9, and lactoferrin), 2 T cell–associated biomarkers (IL-2, IL-10), as well as sST2 and CXCL9 (Figure 7). The biomarker whose 1-log increase in its expected value was associated with the greatest fold increase in the hazard of death was IL-15, with a 14.1-fold increase (4.8 to 45.5), followed by IL-2, MCP-1/CCL2, sST2, NGAL, sTNFRSF1A, CXCL9, MMP-9, IL-10, and lactoferrin (Figure 7 and Supplemental Table 6). IL-1α was the only biomarker associated with a statistically significant decrease in mortality, where a 1-log increase in its expected value was associated with a relative reduction of 80% in the hazard of death (50%–90%). No biomarkers associated with inflammasome activation, or Th1, Th2, or Th17 immune responses, were significantly associated with patient survival in this longitudinal analysis (Figure 7 and Supplemental Table 6).

When comparing the levels of these 11 biomarkers longitudinally in the subset of patients with critical disease during ICU admission and later on in the same patients when they recovered from infection and exited from the ICU to the regular hospital ward, the T cell–associated biomarkers IL-2 and IL-10, the sepsis biomarker sST2, and the monocyte/macrophage activation–associated biomarker IL-15 declined upon patient recovery (Supplemental Figure 15). In contrast, levels of MCP-1/CCL2, NGAL, sTNFRSF1A, CXCL9, MMP-9, lactoferrin, and IL-1α did not significantly change in patients between critical disease in the ICU and upon infection recovery post-ICU (Supplemental Figure 15).

### sST2, sTNFRSF1A, IL-10, and IL-15 may differentiate between survivors and patients who die from COVID-19 when measured throughout the entire hospitalization.

**Figure 8:**
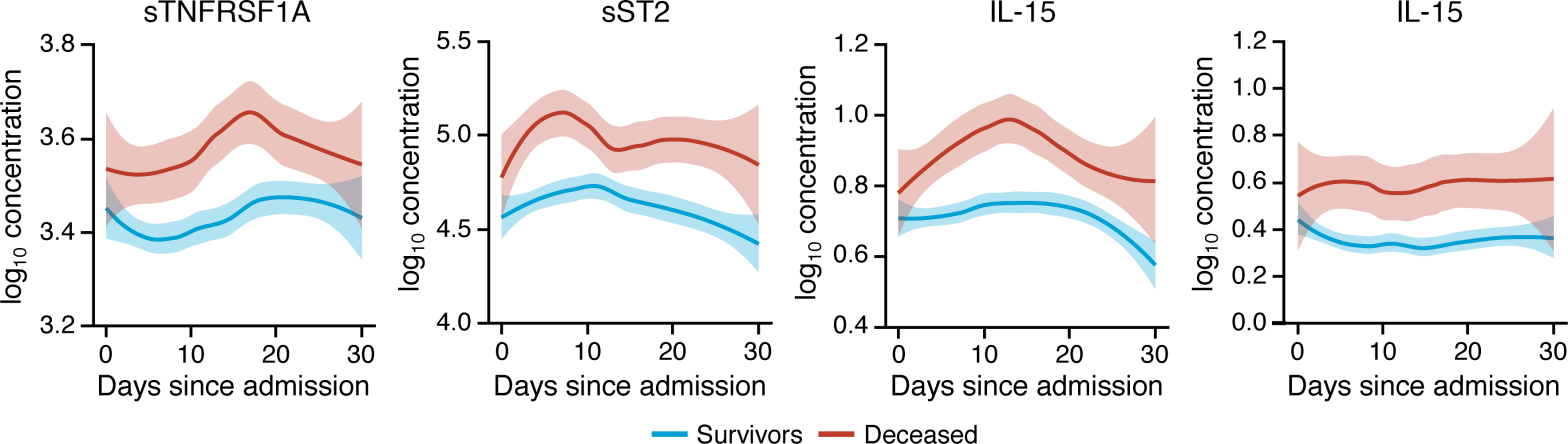
sTNFRSF1A, sST2, IL-10, and IL-15 may differentiate between survivors and patients who succumb to COVID-19 throughout the entire hospitalization. Shown are loess-smoothed means with 95% CIs (shaded intervals) of sTNFRSF1A, sST2, IL-10, and IL-15 concentration throughout the hospitalization in patients with COVID-19 who survived or succumbed to the infection (*n* = 175). All biomarker concentrations are in pg/mL.

Among the 14 identified biomarkers whose longitudinal trajectories were associated with mortality during COVID-19, we noted that sTNFRSF1A, sST2, IL-10, and IL-15 exhibited longitudinal trajectories that clearly segregated survivors versus patients who succumbed to COVID-19 throughout the entire course of hospitalization (Figure 8). All other 62 tested biomarkers exhibited varying degrees of overlap in their longitudinal trajectories between survivors versus patients who died from COVID-19 during hospitalization (Supplemental Figure 16). This indicates that sTNFRSF1A, sST2, IL-10, and IL-15 levels measured at any time during hospitalization, not just within the first few days of admission, might help identify patients at risk for death.

## Discussion

In this study, we have analyzed blood levels of a large number of immune function–related proteins, with the intent to better characterize the inflammatory response of COVID-19 in hospitalized patients and to identify novel biomarkers that may help ascertain clinical outcome. Our data (a) confirm that COVID-19 is characterized by a broad inflammatory signature, with increased levels of soluble biomarkers indicative of activation of various immune cell types, including monocyte/macrophages, neutrophils, T lymphocytes, and nonhematopoietic cells, such as endothelial and epithelial cells; (b) provide novel insights into the immunologic effectors that may contribute to the immunopathogenesis of COVID-19; and (c) suggest that certain immune-based biomarkers, measured either early upon patient admission or throughout hospitalization, may indicate an increased risk for mortality in infected patients.

The relative roles of abnormal IL-1β, NF-κB–driven, and type I/III and type II IFN responses in the immunopathology of severe COVID-19 remain controversial (4, 10, 14, 15, 17). In this study, levels of IL-1β, IL-1RA, IL-18, and IL-18BP were higher in patients with COVID-19 than in HVs; however, they were lower than in patients with canonical autoinflammatory diseases. Moreover, no correlation was observed between levels of these biomarkers and the severity of COVID-19, and no association was found between the initial levels or longitudinal trajectories of these biomarkers with patient mortality. Together, these data suggest a limited contribution of the IL-1β and IL-18 pathways and inflammasome activation to COVID-19–associated clinical outcome.

By contrast, our data indicate a prominent role for NF-κB activation in the progression of the disease. In particular, after adjusting for confounding factors, increased MCP-1/CCL2 and sTNFRSF1A levels in a patient’s initial sample were associated with one of the highest risks of death among all 66 tested biomarkers. Moreover, an NF-κB score based on whole-blood transcriptional levels of 11 NF-κB–regulated genes was markedly elevated in patients with COVID-19 compared with HVs. Finally, concordance between increased levels of some NF-κB–dependent biomarkers (MCP-1/CCL2, MIP-1α/CCL3, IL-6, and sTNFRSF1A) and the NF-κB–associated transcriptional score in whole blood strongly suggests that circulating hematopoietic cells significantly contribute to the systemic inflammatory response during COVID-19.

In our series, upregulation of IFN-γ and CXCL9 levels and increased whole-blood IFN-γ–associated transcriptional score were detected, indicating enhanced IFN-γ responses in COVID-19. By contrast, conflicting results have been reported in the literature on type I/III IFN responses. In particular, Hadjadj et al. (10) identified an impaired type I IFN response accompanied by high viral loads and an excessive NF-κB–driven inflammatory response in COVID-19 patients with severe and critical disease phenotypes. Moreover, impaired induction of type I/III IFN–dependent genes has been reported in postmortem analysis of lungs from COVID-19 patients, and low to undetectable serum levels of IFN-β and IFN-λ have been detected in SARS-CoV-2–infected individuals (7). By contrast, others have reported heightened type I IFN responses in the respiratory tract, and robust albeit not uniform expression of IFN stimulated genes in circulating monocytes, of COVID-19 patients (43, 74). Our data have revealed dissociation between increased blood IFN-α2a levels and enhanced type I IFN–associated transcriptional scores in whole blood and low *IFNA2* transcriptional levels in whole blood from patients with COVID-19. These observations suggest that the major source of IFN-α2a (and by inference, of other type I IFNs) is not primarily represented by circulating blood cells but most likely by tissue-resident cells, possibly virus-infected lung epithelial cells. Similar results have been recently reported by others (16). Although no correlation was observed between IFN-α2a levels and disease severity or risk of death in the patients analyzed in our study, we have evidence that impaired type I IFN production or signaling may contribute to aggravate the clinical phenotype of COVID-19 in patients with monogenic errors of type I IFN-mediated immunity or with neutralizing anti–type I IFN antibodies (75, 76).

Furthermore, our data imply an important role of neutrophils in COVID-19 pathophysiology and disease progression. In particular, neutrophils displayed extensive vacuolization consistent with an activated state, and blood levels of 3 neutrophil-derived molecules (MMP-9, NGAL, and S100A9) showed positive correlation with the risk of death, while the longitudinal trajectory of 3 neutrophil-derived molecules (NGAL, MMP-9, lactoferrin) was also associated with the risk of death. A previous study showed that neutrophils play a critical role in the development of ARDS in patients with COVID-19 (77), and a recent study showed that administration of G-CSF to neutropenic COVID-19 patients was associated with a rise in absolute neutrophil count and an increased risk of respiratory failure and death (78). Neutrophils are also known to play a key role in triggering sepsis-associated ARDS (79). This is especially interesting in light of our multivariable and longitudinal data showing that the sepsis biomarker sST2 was associated with mortality and with the observation that sST2 levels declined during recovery of patients who had required prior admission to the ICU. Of interest, a recent report found increased levels of LPS in the plasma of patients with severe COVID-19 (16); as such, the increased levels of sST2 identified in our study may reflect bacterial or bacterial pathogen-associated molecular pattern translocation during severe SARS-CoV-2 infection.

Besides sTNFRSF1A, IL-15, sST2, and IL-10 emerged as the 3 other biomarkers whose blood levels clearly distinguished survivors from nonsurvivors during the entire length of hospitalization. IL-15 is a pleiotropic cytokine predominantly expressed by monocytes/macrophages and dendritic cells but also by nonhematopoietic cells such as keratinocytes and epithelial cells (80). Although IL-15 has been ascribed a major role in NK cell development and function, it is also involved in inflammatory responses. In particular, IL-15 promotes the production and secretion of IL-8 by neutrophils and stimulates their migration, thereby contributing to their recruitment at inflammatory sites (81). In a previous study, increased serum levels of IL-15 were present in patients with early ARDS who died as compared with those who survived, whereas the opposite was true for IL-15 levels in bronchoalveolar lavage fluid (82). In another study, IL-15 serum levels correlated with disease severity in children with bronchiolitis (83). IL-15 immunotherapy, in the intent to potentiate T cell and NK cell antiviral responses, has been advocated as a potential viable strategy for COVID-19 (84); the results obtained in our study raise caution on this approach.

sST2 serves as a soluble decoy receptor for IL-33, and its expression is enhanced by proinflammatory cytokines in human lung epithelial cells (85), in particular when neutrophilic inflammation is present (86–88). Moreover, sST2 has previously been shown to predict disease severity in children with acute viral lower respiratory tract infections (89). Together, these studies further suggest a prominent role for neutrophilic lung inflammation as a major contributor to poor outcome in COVID-19.

Moreover, multiple studies have reported increased IL-10 blood levels, correlating with disease severity and progression, in patients with COVID-19 (4, 11, 15, 19, 45). The cell source and specific effects of increased IL-10 in patients with severe COVID-19 remain to be defined. However, it is interesting to note that IL-10 inhibits expression of HLA class II molecules by antigen-presenting cells, a phenomenon that has been observed in myeloid cells of patients with severe COVID-19 (16, 40, 90, 91). Furthermore, a significant increase in IL-10–producing regulatory T cells has been observed in the blood of patients with severe COVID-19, compared with those with moderate and mild disease and HVs (92). In mouse models, IL-10–producing regulatory T cells play a critical role in controlling lung inflammation by restraining development of tissue-damaging Th17 cells and inhibiting innate inflammatory responses (93, 94). The higher IL-10 levels detected in our study in patients with COVID-19 who died may have reflected an extreme attempt to counteract severe lung inflammation. On the other hand, it is also possible that increased IL-10 may suppress antiviral adaptive immune responses and weaken resistance to bacterial superinfections in COVID-19 patients, as previously shown in animal models of influenza infection (95, 96). Notably, IL-10 and sTNFRSF1A were among the biomarkers most strongly associated with mortality in our analysis. Prior studies have demonstrated that IL-10 can upregulate the expression of sTNFRSF1A, which raises the question as to whether these proteins are coregulated in COVID-19 (97, 98).

A surprising finding in our analysis was the association of decreased longitudinal trajectories of IL-1α with increased risk of death in COVID-19 patients. It remains elusive how decreased levels of IL-1α, a danger-associated molecular pattern typically released from injured epithelial cells, might heighten the risk of death during COVID-19. Thus, future studies are required to validate and help interpret these results.

Our study has several limitations. The patient population had a skewed representation of disease phenotypes, with a predominance of patients with severe and critical disease, consistent with the severe evolution of COVID-19 in northern Italy at that time. In addition, it was not feasible to obtain healthy donor samples from Italy during the peak of the pandemic; therefore we analyzed North American healthy donors’ samples. Studying patients with other forms of infectious and noninfectious interstitial pneumonitis may help define to what degree the abnormalities observed in this study are specific to SARS-CoV-2 infection. In the longitudinal model, the association between mortality and each biomarker was described in terms of the expected change in the instantaneous hazard of death per unit change in the concurrent expected value of the biomarker, with the expected value of the log biomarker modeled as a population-level linear trend, offset by a subject-specific intercept parameter. Consequently, while the longitudinal model captured whether an individual’s average biomarker level was above the population mean, as it might be if that person had experienced cytokine storm at some point, it did not contain the sort of granular time-varying covariates that could be used to identify motifs reflecting increased biomarker expression since no such covariates were available. From our perspective, the collection of temporally dense granular immunologic data could help to shed light on when and why individuals entered into different immunologic states that would be reflected in the biomarkers whose average values over the observation period were identified as being associated with mortality. Notwithstanding these limitations, we have provided a systematic analysis of a large number of soluble inflammatory biomarkers, described their trajectory over time, and analyzed the correlation of their initial levels and longitudinal evolution with the risk of death from COVID-19 after adjusting for possible confounding factors.

In summary, we have identified potential biomarkers of COVID-19 severity that provide novel insights into the complex immunopathogenesis of COVID-19. If validated in independent cohorts of patients, these results may help identify COVID-19 patients who are at risk of mortality and in whom individualized strategies for risk assessment and therapeutic intervention might improve their outcomes.

## Methods

### Patient enrollment and determination of COVID-19 illness severity.

Deidentified patient plasma and serum samples were obtained from discarded, clinically indicated collection of blood samples obtained from 175 patients admitted at ASST Spedali Civili Brescia, Italy, Ospedale San Gerardo, Monza, Italy, and Ospedale S. Matteo, Pavia, Italy, following positive nasopharyngeal swab PCR (*n* = 173) or positive serology for SARS-CoV-2 infection (*n* = 2) (99). Patients’ clinical information and eligibility were surveyed with the standard COVID-19 Human Genetic Effort Patient Screen form. Severity of COVID-19 disease for each patient was ascertained per the Diagnosis and Treatment Protocol for Novel Coronavirus pneumonia (trial version 7), released by the National Health Commission & State Administration of Traditional Chinese Medicine on March 3, 2020 (21).

Blood samples from American HVs were drawn after written informed consent was obtained under the Frederick Research Donor Program protocol OH99-C-N046. The mean age of HVs was 44.9 years (range, 25.2–71 years old), 53.4% were male, 46.6% were female, and no HVs had comorbidities (Supplemental Table 7). Blood samples from patients with autoinflammatory conditions were obtained under the NIH IRB-approved protocol NCT02974595. Hospitalized COVID-19 patients at the NIH Clinical Center or George Washington University Hospital were enrolled in NIH IRB-approved protocols NCT00001467 and NCT01200953 for cytometry-based analyses. HVs were enrolled in NIH IRB-approved protocol NCT01386437 for cytometry-based analyses. Study participants provided written informed consent in accordance with the Declaration of Helsinki.

Detailed methods for measurement of soluble biomarkers, flow cytometric studies in whole blood of COVID-19 patients, preparation of peripheral blood smears, transcriptional analysis of whole blood from PAXgene tubes, and additional statistical methods are available in Supplemental Methods.

### Definition of clinical outcomes used for survival modeling.

Mortality was defined as death within 6 weeks of hospital admission. For patients with multiple hospitalizations, the date of the patient’s index hospitalization was used as the start date to define the 6-week interval. Data from patients whose status was unknown at 6 weeks after hospital admission were censored on the date the patient was last known to be alive.

### Survival models fit to first sample for each biomarker.

To start, we fit a Cox proportional hazards (PH) model to the first biomarker measurement for each patient, adjusting for time from admission to sample collection. Recognizing that survival may be affected by a variety of factors that are associated with different biomarkers, we extended this first model to additionally adjust for multiple confounders, including age, receipt of immunomodulatory medications, and history of chronic kidney disease, which were found to be associated with alteration in a large number of candidate biomarkers (Supplemental Table 5). For these models, we report the aHR and 95% CI associated with a 1-log increase in biomarker concentration. As a supplementary analysis, we also fit an unadjusted PH model to biomarker measurements that were collected within the first 7 days postadmission (see Supplemental Methods).

### Joint longitudinal–survival model fit to all biomarker samples.

To assess whether mortality was associated with the expected value of the biomarker over time, we used all samples from 175 patients, regardless of when the first sample was drawn during their hospitalization. Biomarkers were measured repeatedly in 144 patients (82%), with 98 patients (56%) having 3 or more longitudinal samples, for a total of 609 distinct samples. Ferritin measurements were only available for 123 patients.

### Joint model.

We used shared parameter joint models to describe trends in each biomarker over time and the association between the biomarker and a patient’s risk of death. These models combine a longitudinal mixed effects model fit to repeated measurements of a biomarker with a survival model estimating time to death (72, 73). Joint modeling in this analysis was conducted within a Bayesian framework using the rstanarm package (100, 101). For the longitudinal submodel, we specified a generalized linear mixed model, with fixed effects for days from hospital admission to sample collection, and subject-specific intercept parameters to account for repeated samples within patients. Time to death was estimated using a PH submodel with the expected value of the biomarker estimated by the longitudinal submodel included as a covariate. The survival submodel also adjusted for age, history of chronic kidney disease, and whether the patient received immunomodulatory medications. The baseline hazard was estimated using B-splines. Weakly informative priors were used on all parameters except for the association parameter, which was set so that 95% of the prior mass fell between a 90% relative reduction hazard and a 10-fold relative increase in the hazard. Additional details regarding the priors and the model fitting procedure are provided in Supplemental Methods. The posterior median aHR and 95% credible intervals are reported, as are posterior survival probabilities and longitudinal biomarker trajectories summarizing the posterior predictive distribution.

### FDR control.

We decided whether an association between a biomarker and survival was deemed statistically significant based on FDRs estimated from 1-sided *P* values, or posterior error probabilities in the case of the joint model, of tests for whether a given biomarker was positively or negatively associated with mortality. We set our significance thresholds to separately control the FDRs for each model at 0.025 for each of the 2 sets of 1-sided comparisons (102, 103).

### Imputation of missing data.

Missingness in covariate and biomarker values are tabulated by sample and patient in Supplemental Tables 9 and 10, respectively. Missing data were imputed using multiple imputation by chained equations via the mice package in R (104). A particular biomarker measurement was considered missing on a given day if the patient was tested for some, but not all, biomarkers that day. Biomarker concentrations were log-transformed prior to imputation. We generated 50 imputed data sets using classification and regression trees across all variables (see Supplemental Methods for more information), with each imputation based on 20 sampling iterations, which was deemed sufficient for convergence of the imputation algorithm based on trace plots of imputed values. Reported estimates were based on the pooled fits for each of the 50 imputed data sets.

### Statistics.

We described the association between a patient’s risk of death and level of each biomarker using 3 survival models that increased in the scope of the data that was incorporated into the model (Supplemental Table 8). For each biomarker, groupwise median ± IQR was calculated (Supplemental Table 3). For comparisons of continuous variables between 2 groups, a 2-tailed unpaired Student’s *t* test was used. Significance was defined as *P* < 0.05. Comparisons involving more than 2 groups were made using the Kruskal-Wallis test. When *P* < 0.05, pairwise comparisons were made using Dunn’s test with Benjamini-Hochberg adjustment for multiple comparisons variables. Significance was defined as adjusted *P* < 0.05. For survival modeling, significance was defined as *q* < 0.025 (see Supplemental Methods for more details).

### Study approval.

Ethical approval was obtained from the University of Milano-Bicocca School of Medicine, San Gerardo Hospital, Monza – Ethics Committee of the National Institute of Infectious Diseases Lazzaro Spallanzani (protocol 84/2020, COVID-STORM); the IRB of Fondazione IRCCS Policlinico San Matteo, Pavia (protocol 20200037677); and the Comitato Etico Provinciale, Brescia, protocol NP 4000, CORONAlab. Written consent was waived as all testing was performed on discarded blood specimens collected during the course of clinical practice. Blood samples from American HVs were drawn after written informed consent was obtained under the Frederick Research Donor Program protocol OH99-C-N046. Blood samples from patients with autoinflammatory conditions were obtained under the NIH IRB-approved protocol NCT02974595. Hospitalized COVID-19 patients at the NIH Clinical Center or George Washington University Hospital were enrolled in NIH IRB-approved protocols NCT00001467 and NCT01200953 and HVs were enrolled in NIH IRB-approved protocol NCT01386437 for cytometry-based analyses. Study participants provided written informed consent in accordance with the Declaration of Helsinki.

## Author contributions

MSA, OMD, EER, and JF equally contributed. Author order was determined by the overall contribution of these authors to the study. JIG, DBK, RGM, MSL, and LDN equally contributed. MSA, OMD, EER, JF, JIG, DBK, RGM, MSL, and LDN contributed to study concept and design. MSA, DLF, KD, VO, JVD, DM, AS, AAADJ, DEK, EFG, ATR, and KAZ processed the samples and performed the experiments. MSA, OMD, ES, LI, EQR, LRB, MD, RC, HCS, MS, and KMH collected the data. MSA, EER, JF, AAADJ, SA, OMD, VO, JVD, DLF, JF, KRC, and BS analyzed the data. EQR, LI, LRB, MD, GLM, RC, FC, CR, AB, PB, DB, DWC, and AL provided patient samples. LDN, DBK, RGM, MSL, SWC, HCS, PDB, JIC, HCS, JST, SLL, MJD, and JIG provided reagents, materials, and analysis tools. MSA, MSL, and LDN wrote the manuscript. All authors read and approved the final manuscript.

## Supplementary Material

Supplemental data

Supplemental Table 3

Supplemental Table 4

Supplemental Table 5

## Version 1 11/24/2020

In-Press Preview

## Version 2 01/11/2021

Electronic publication
