# Supplementary material for: An immune-based biomarker signature is associated with mortality in COVID-19 patients
Source: JCI Insight. 2021 Jan 11;6(1):e144455. doi: 10.1172/jci.insight.144455 (PMC7821609; doi:10.1172/jci.insight.144455)
Supplement: Supplemental data [file jciinsight-6-144455-s049.pdf]

Supplementary Materials and Methods

Measurement of soluble biomarkers

Cytokine, chemokine and other biomarker analysis was performed on EDTA plasma or sera obtained from COVID-19 patients and healthy volunteers (HV). Because of limited available volume, patient samples were analyzed as single determinations. Duplicate determinations of control samples and samples from HV yielded coefficients of variation that were normally <20%. Blood samples were centrifuged, and serum or plasma samples frozen immediately in a -85ºC freezer prior to analysis, which occurred within 1-4 weeks (for COVID-19 patients) or up to 1 year (HV) of blood collection. Cytokines (IL-1α, IL-1β, IL-2, IL-4, IL-5, IL-6, IL-7, IL-8, IL-10, IL-12p70, IL-12p40, IL-13, IL-15, IL-16, IL-17, IFN-γ, TNF-α, TNF-β, GM-CSF, VEGF, CCL-11/Eotaxin-1, CCL26/Eotaxin-3, CXCL10/IP-10, MCP-1/CCL2, MCP-4/CCL13, CCL22/MDC, MIP-1α/CCL3, MIP-1β/CCL4, CCL17/TARC) were measured using the V-PLEX Human Cytokine 30-Plex Kit (Meso Scale Discovery, Rockville, MD) an analyzed on a MESO QuickPlex SQ 120 reader (Meso Scale Discovery, Rockville, MD) according to the manufacturers specifications. IFN-α2a was determined on a single analyte, ultra-sensitive S-PLEX IFN-α2a kit (Meso Scale Discovery, Rockville, MD) according to the manufacturer's specifications. Additional cytokines (IL-1RA, IL-3, IL-23, IL-33, G-CSF, M-CSF, CX3CL1/Fractalkine, sCD40 ligand/TNFSF5, soluble FAS ligand/TNFSF6, TNFSF14/LIGHT, SCF/c kit ligand), and other soluble receptors/biomarkers (sTNFRSF1A/sTNF RI, sTNFRSF1B/sTNF RII, sST2/sIL-33R, sCD25/sIL-2Rα, sICAM-1/sCD54, sVCAM-1/sCD106, sCD31/sPECAM, sL-selectin/sCD62L, sE-

selectin/sCD62E, RAGE, sCD163, sVEGFR1/Flt-1, REG3A, S100A8, S100A9, MMP-9, lactoferrin, MPO, lipocalin-2/NGAL, LBP) were measured on customized, magnetic bead-based, multiplex assay (R&D Systems, Minneapolis, MN) according to the manufacturers specifications for standards and dilutions. The magnetic beads were analyzed on Bio-Plex 3D instrumentation (Bio-Rad, Hercules, CS). Standard curves were analyzed using nonlinear curve fitting and unknowns were calculated based on the derived equation. Samples that exceeded the highest standards were reanalyzed more dilute until the values fell within the range of the known standards. Two control plasma samples and a control sample spiked with a known quantity of each analyte were analyzed on each plate to assess the inter-plate variation and to determine the effect of the biological matrix on the measurement of each analyte. For most analytes, the control samples had <25% variation from plate to plate, and the recoveries were generally >70%.

Ferritin was measured in the clinical laboratories of the hospitals where patients were admitted. CXCL9 levels were measured using a Duoset ELISA kit (R&D Systems). Total IL-18 and IL-18BP levels were measured as previously described (1). Briefly, serum was diluted 25-fold and assayed on a FLEXMAP 3D multiplex instrument per the manufacturer’s instructions (Luminex). Recombinant IL-18 was used as standard (MBL International), and human IL-18BPa-Fc (R&D Systems) was run as a separate standard for IL-18BP. IL-18 and IL-18BPa beads were generated by conjugating capture antibody to magnetic beads per the manufacturer’s instructions (Bio-Rad). All reagents used were derived from the same lots. Minimal variation between plates and runs was verified using bridging controls.

To measure pGSN, Nunc Maxisorp plates were coated with 100 µl of 5 µg rabbit anti-human pGSN pAb specific for the 24 amino acid extension unique to plasma gelsolin/ml 0.05M carbonate buffer pH 9.6 for 2 hr at room temperature. After washing 2x in wash buffer (150 mM NaCl, 25 mM Tris 7.4, 1 mM $CaCl_2$, 0.05% Tween-20) plates were incubated in the wash buffer + 3% BSA for 10 minutes to block. Samples and recombinant standard were diluted in wash buffer + 1% BSA and incubated with detection buffer (wash buffer + 1% BSA with 0.4 µg mouse anti-gelsolin mAb clone GS-2C4 (Sigma) and 0.067 µg Goat anti-Mouse IgG (H+L) – HRPO (eBioScience/Invitrogen) for 30 min at room temperature with shaking. After washing 4x, 100 µl of TMB substrate (Invitrogen) was added per well and allowed to develop for 5 minutes before stopping with 100 µl 2 N H_2SO_4 and read at A_{450} and A_{650} prior to concentration determination using a 5-parameter logistic curve (GraphPad Prism 8).

Paired serum and EDTA plasma samples drawn concurrently from HV (n = 15) and patients with COVID-19 (n = 25) were analyzed for all biomarkers. The paired data were plotted, and linear regression lines were determined. If the slope of the regression line for a particular biomarker fell between 0.6 and 1.4, the level in EDTA plasma and serum were considered equivalent and either serum or plasma was used for analysis. For all other biomarkers that the slope of the regression line did not fall within the above range (MCP-1/CCL2, TNFSF14, VEGF, NGAL, MPO, MMP-9, S100A9, lactoferrin, IL-8, sCD40L, IL-7, CXCL10, IL-13, CCL11, CCL13, CCL17, and CCL26), only serum was used for testing (Supplementary Figure 17).

Flow cytometric studies in whole blood of COVID-19 patients

COVID-19 patient and HV whole blood samples were harvested in the morning and were processed for flow cytometry-based analyses within 3 hours of blood harvesting. In order to assess expression of cell surface markers indicative of myeloid cell activation, heparinized whole blood was first treated with RBC Lysing Buffer (BD Biosciences). Subsequently, the LIVE/DEAD™ Fixable Blue Dead Cell Stain Kit (Thermo Fisher) to stain dead cells. In order to prevent non-specific antibody binding, the cells were treated with human FcR Blocking Reagent (Miltenyi) and 0.5% bovine serum albumin (Sigma). The myeloid cells and corresponding activation markers were stained using antibodies against human CD11b (clone eBio ICRF44; Thermo Fisher), CD45 (clone HI30; Biolegend), CD16 (clone 3G8; BD Biosciences), CD18 (clone 6.7; BD Biosciences), CD169 (clone 7-239; Biolegend), CD63 (clone H5C6; Biolegend), b558 (clone 7D5; MBL Life science), CD66b (clone G10F5; Biolegend) and CXCR2 (clone eBio5E8-C7-F10; Thermo Fisher). The stained cells were analyzed using LSRFortessa flow cytometer (BD). The data files from flow cytometer were exported and analyzed using FlowJo (BD Biosciences) and Excel (Microsoft), while plots were created in Prism (Graphpad).

In order to assess intracellular IFN-γ, IL-4 and IL-17A, heparinized unstimulated whole blood was processed as described previously (2). Different lymphocyte populations were stained using antibodies against human CD3 (clone SK7; Thermo Fisher), CD4 (clone RPA-T4; BD Biosciences), CD8 (clone SK1; Biolegend), CD56 (clone NCAM16.2; BD Biosciences) and CD19 (clone HIB19; Biolegend), while antibodies against human IFN-γ (clone 4S.B3; Biolegend), IL-17A (clone eBio64CAP17; Thermo Fisher) and IL-4 (clone MP4-25D2; Biolegend) were used to stain for

intracellular cytokine after fixation and permeabilization as described previously (2). The stained cells were analyzed using LSRFortessa flow cytometer (BD). The data files from flow cytometer were exported and analyzed using FlowJo (BD Biosciences) and Excel (Microsoft), while plots were created in Prism (Graphpad).

Analysis of peripheral blood smears

Routine peripheral blood smears were prepared using a Sysmex SP-10 automated slide maker stainer. Slides were scanned and cells were classified using a CellaVision DM96 instrument. CellaVision images were examined for monocyte and neutrophil morphology from seven COVID-19 patients hospitalized at the NIH Clinical Center. At least 100 monocytes and 100 neutrophils were evaluated per patient to determine the percent of each cell type that contained vacuoles.

Transcriptional analysis of whole blood from PAXgene tubes

Eighty-four COVID-19 patients had serial whole blood collections in PAXgene tubes; 11 (13%) had moderate disease, 8 (9%) had severe disease, 45 (54%) had critical disease and 19 (23%) ultimately succumbed to their disease. Total RNA was extracted from whole blood samples collected in PAXgene tubes (Qiagen, Germantown, MD) and subjected to transcriptional analysis of selected genes including *IFNA2* was determined by NanoString (NanoString Technologies, Seattle, WA). A 28-gene type I IFN score and an 11-gene NF-κB score were calculated as previously described (3, 4). An IFN-γ score was calculated based on 15 IFN-γ-regulated genes (5). Briefly, the 28-gene type I IFN score is the sum of the z-scores of 28 type I IFN response genes and the 11-gene NF-

κB score is the sum of the z-scores of 11 NF-κB targets genes and the 15-gene IFN-γ score is the sum of the z-scores of 15 IFN-γ response genes. Individual gene z-scores were calculated using the mean and standard deviation of the NanoString counts from 22 HV (Supplementary Table 11).

Peripheral blood smear examination

Routine peripheral blood smears were prepared and wer examined for monocyte and neutrophil morphology from seven COVID-19 patients hospitalized at the NIH Clinical Center. At least 100 monocytes and 100 neutrophils were evaluated per patient to determine the percent of each cell type that contained vacuoles.

Statistical analyses

The effect of age (≥65 years), gender, and various medical comorbidities on biomarker concentration was assessed by a Mann-Whitney U test. P-values were adjusted for multiple comparisons using the Benjamini-Hochberg method. For comparisons between HV and all COVID-19 patients, an unpaired Student's t-test or a Mann-Whitney U test was performed depending on whether the data were normally distributed. For all comparisons involving >2 groups (including the comparisons between HV and various COVID-19 severity groups), a Kruskal-Wallis test was performed. When $P<0.05$, pairwise comparisons were made using Dunn's test with Benjamini-Hochberg adjustment for multiple comparisons. The correlation between normalized *IFNA2* gene counts and IFN-α2a concentration or type I IFN gene score were determined by Spearman’s rank correlation test. Nonparametric two-tailed

Kruskal-Wallis test was used to compare transcriptional scores for group comparisons and *P* values <0.05 were considered statistically significant. Statistical analyses were performed using GraphPad Prism version 8.

Prior specification and computational details for the joint longitudinal-survival model

We fit the shared parameter joint longitudinal-survival models to each of the imputed datasets via Hamiltonian Monte Carlo via the rstanarm package (6, 7). We combined posterior samples from five Markov chains, each run for 5000 iterations with the first 2500 iterations discarded as warmup, leaving an overall posterior sample size of 12500 samples per fit. The posterior samples from all 50 fits were then combined to obtain a combined sample from the joint posterior distribution of the model parameters and missing data. Convergence within each fit was assessed by checking all scale reduction factors were <1.01. We set weakly informative priors, specified in the table below. The covariate parameters in the longitudinal model describe the change in the expected value of the log transformed biomarker per unit change in the covariate. Covariates parameters in the event model describe the expected change in log-hazard per unit change in the covariate. The association parameter in the event model is interpreted as the expected change in log-hazard per unit change in the expected value of the log biomarker.

Parameter	Prior	Prior median (95% Interval)
Longitudinal model		
Intercept	Normal (0, 10)	0 (-19.6, 19.6)
Covariates	Normal (0, 2.5)	0 (-4.9, 4.9)
Standard deviation of random intercepts	Half Student-t (4, 0, 10)	10 (0.4, 255)
Error standard deviation	Half Student-t (4, 0, 5)	7.4 (0.3, 35)
Event model		
Covariates	Normal (0, 2.5)	0 (-4.9, 4.9)
Association parameter	Normal (0, 1.175)	0 (-2.3, 2.3)
Spline coefficients	Student-t (4, 0, 5)	0 (-13.9, 13.9)

Supplementary Materials and Methods References

1. Weiss ES, et al. Interleukin-18 diagnostically distinguishes and pathogenically promotes human and murine macrophage activation syndrome. *Blood.* 2018;131(13):1442-55.
2. Roschewski M, et al. Inhibition of Bruton tyrosine kinase in patients with severe COVID-19. *Sci Immunol.* 2020;5(48).
3. de Jesus AA, et al. Distinct interferon signatures and cytokine patterns define additional systemic autoinflammatory diseases. *J Clin Invest.* 2020;130(4):1669-82.
4. Kim H, et al. Development of a Validated Interferon Score Using NanoString Technology. *J Interferon Cytokine Res.* 2018;38(4):171-85.
5. Liu SY, Sanchez DJ, Aliyari R, Lu S, and Cheng G. Systematic identification of type I and type II interferon-induced antiviral factors. *Proc Natl Acad Sci U S A.* 2012;109(11):4239-44.
6. Brilleman SL CM, Moreno-Betancur M, Buros Novik J, Wolfe R. Joint longitudinal and time-to-event models via Stan. StanCon 2018. 10-12 Jan 2018. Pacific Grove, CA, USA. *https://githubcom/stan-dev/stancon_talks/.* 2018.
7. Goodrich B GJ, Ali I, Brilleman rstanarm: Bayesian applied regression modeling via Stan. . *R package version 2211* 2020;https://mc-stan.org/rstanarm.

NIAID COVID-19 Consortium

Neha Bansal[1], Jason Barnett[1], Marita Bosticardo[1], Samuel Chauvin[1], Xi Cheng[1], Jeffrey Danielson[1], Elizabeth Garabedian[1], Jingwen Gu[1], Galina Koroleva[1], Vasu Kuram[1], William Lau[1], Zhiwen Li[1], Joseph Mackey[1], Mary Magliocco[1], Helen Matthews[1], Marshall Nambiar[1], Andrew Oler[1], Francesca Pala[1], Smilee Samuel[1], Qinlu Wang[1], Sarah Weber[1], Sandhya Xirasagar[1], Yu Zhang[1]

[1] NIAID, NIH, Bethesda, MD, USA

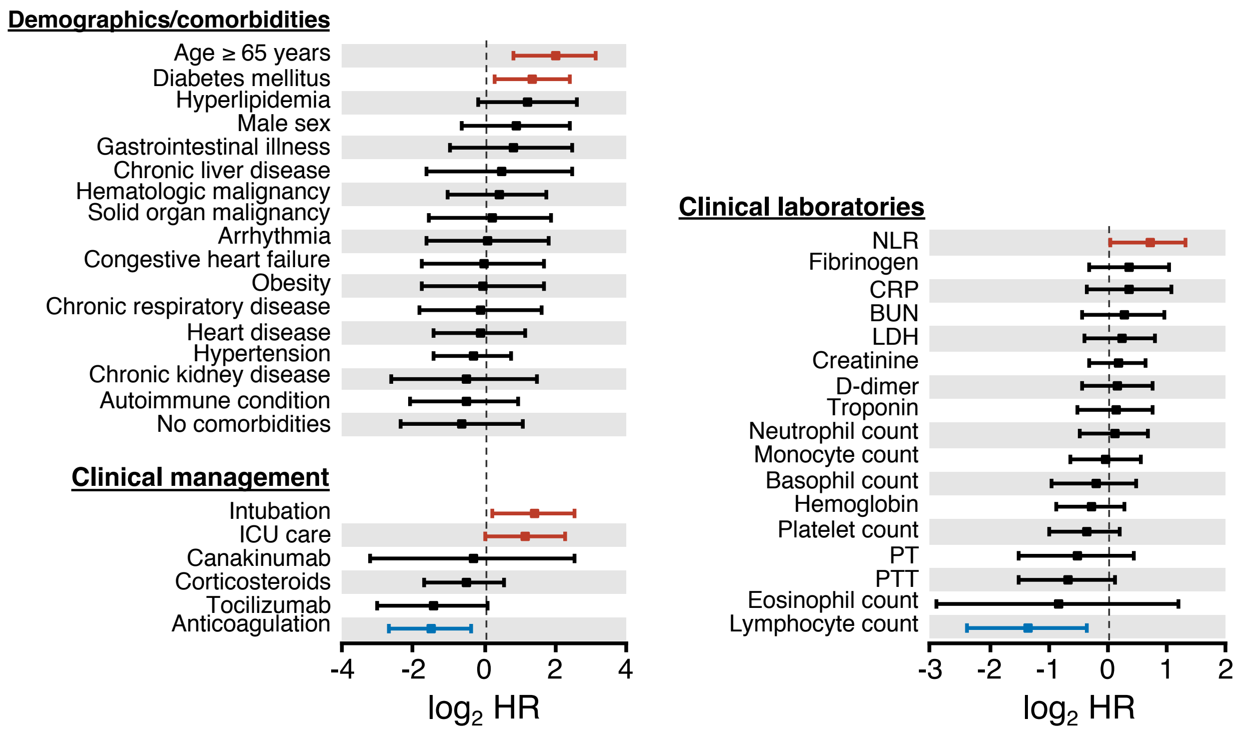

Supplementary Figure 1. **Clinical factors and laboratory tests associated with mortality in COVID-19 patients in our cohort.** Shown are forest plots and HRs by univariable analysis of select clinical factors, therapeutic interventions and laboratory tests and their association with mortality in COVID-19 patients (n = 175). BUN, blood urea nitrogen; CRP, C-reactive protein; ICU, intensive care unit; LDH, lactate dehydrogenase; NLR, neutrophil to lymphocyte ratio; PT, prothrombin time; PTT, partial thromboplastin time. HR confidence intervals for variables significantly associated with mortality (i.e., q-value <0.025) are shown in red when HR >1 and in blue when HR <1. HR confidence intervals for variables with q-values >0.025 are shown in black.

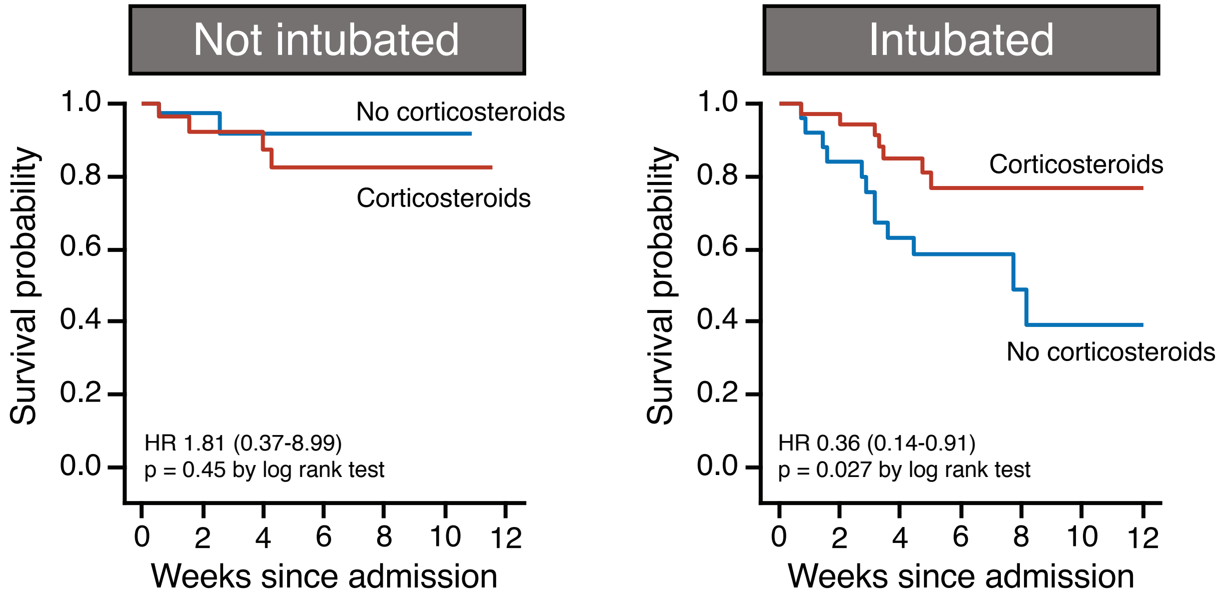

Supplementary Figure 2. **Corticosteroid administration and its association with mortality in intubated and non-intubated COVID-19 patients in our cohort.** Shown are Kaplan-Meier curves showing survival of COVID-19 patients as a function of corticosteroids administration in non-intubated (left panel) (n = 96) or intubated (right panel) (n = 60) individuals in the 156 patients in whom information about corticosteroid administration was available. Shown are HRs, 95% CI values and *P* values calculated by log rank test.

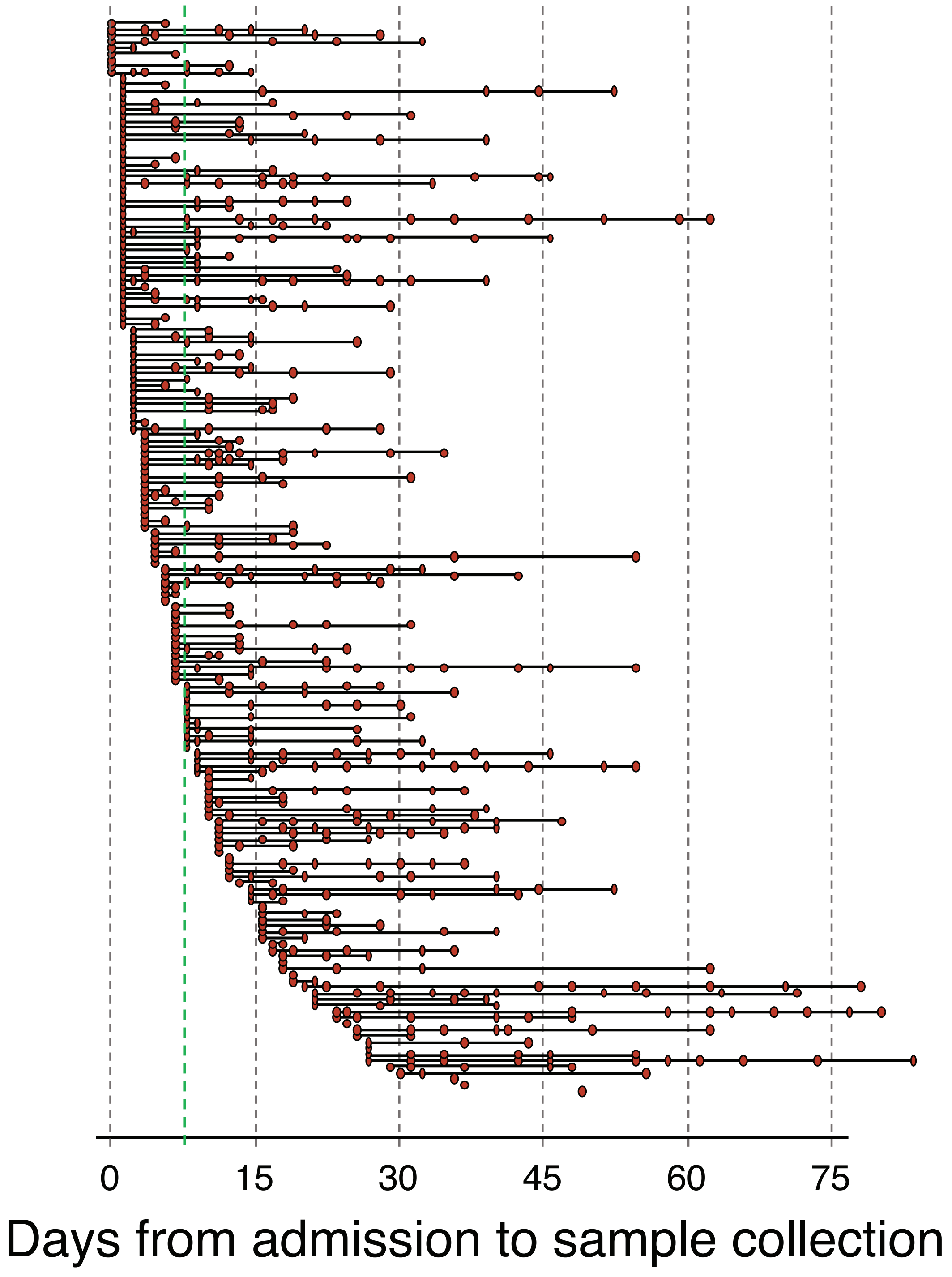

Supplementary Figure 3. Schematic representation of time-points of longitudinal sample collection in the COVID-19 patients of our cohort. Shown are individual time-points of blood harvesting in the 175 COVID-19 patients in our cohort. Each row represents a patient. Each red dot represents an independent time-point that a blood sample was collected, relative to admission, and subjected to biomarker analysis.

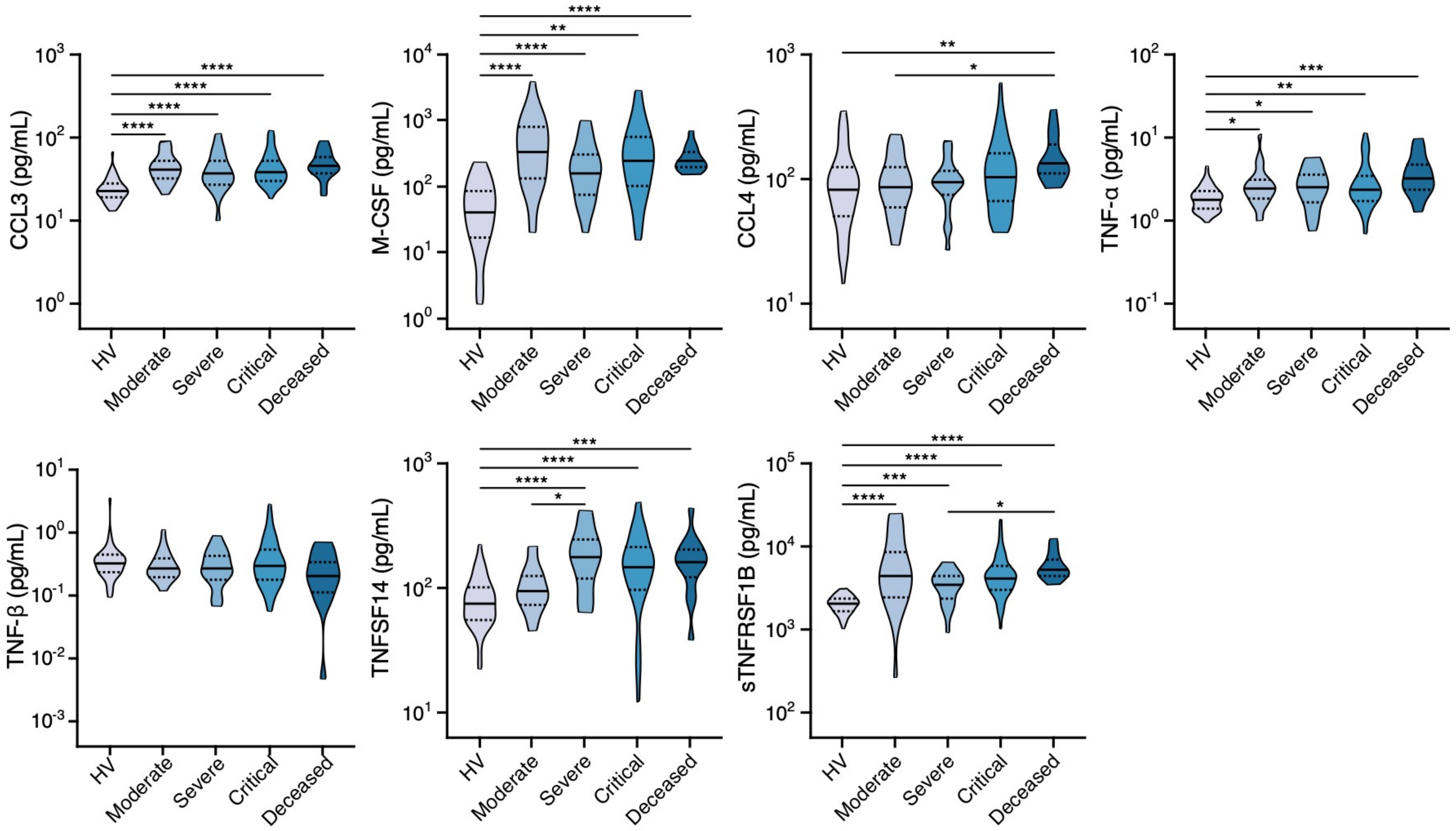

Supplementary Figure 4. **Levels of additional biomarkers associated with activation of monocytes/macrophages and NF-κB signaling in COVID-19 patients.** Shown are levels of CCL3, M-CSF, CCL4, TNF-α, TNF-β, TNFSF14 and sTNFRSF1B in peripheral blood of COVID-19 patients with various severity groups (n = 94-119 depending on the biomarker) relative to healthy volunteers (HV; n = 45-60 depending on the biomarker). Groups were compared by Kruskal-Wallis test. When P<0.05, pairwise comparisons were made using Dunn's test with Benjamini-Hochberg adjustment for multiple comparisons. *P<0.05, **P<0.01, ***P<0.001, ****P<0.0001.

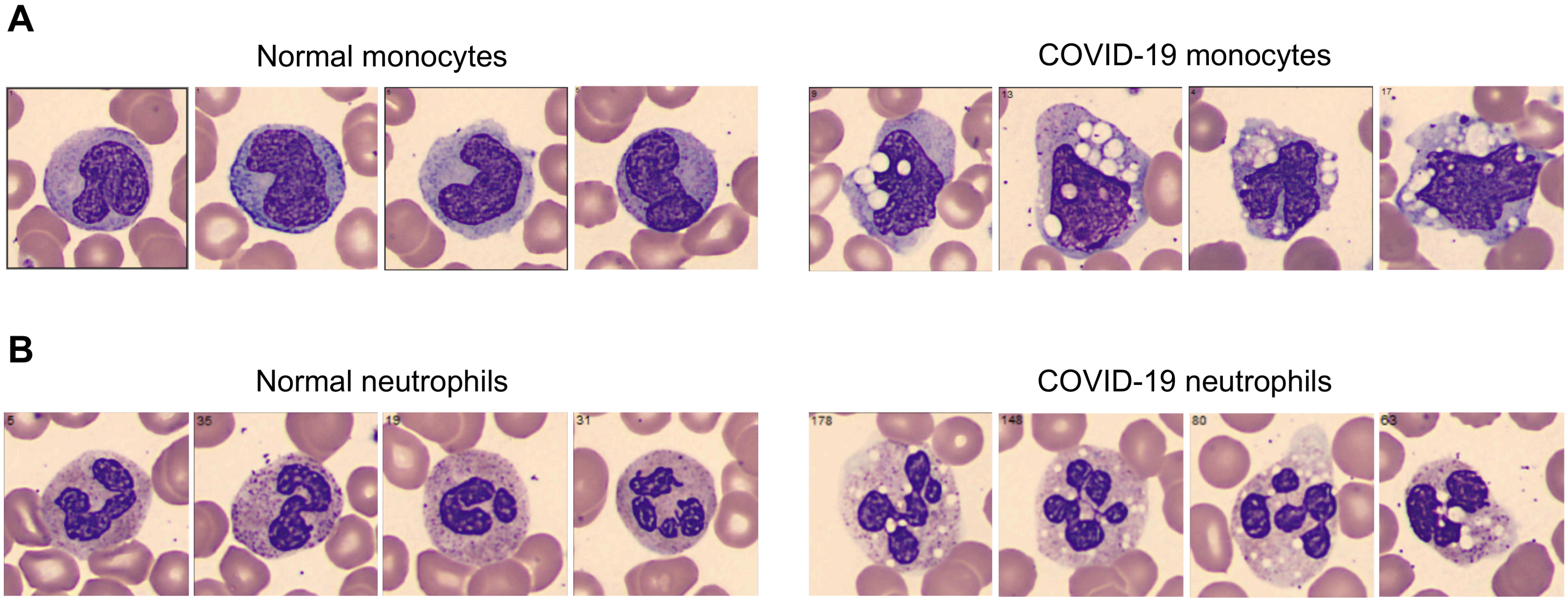

Supplementary Figure 5. Vacuolization in peripheral blood monocytes and neutrophils of COVID-19 patients. Shown are representative peripheral blood smear micrographs showing vacuolization in (A) monocytes and (B) neutrophils from hospitalized hypoxemic COVID-19 patients (n = 7) relative to healthy volunteers. Increased vacuoles were noted in ~80% of monocytes and ~50% of neutrophils in each COVID-19 patient throughout their hospitalization.

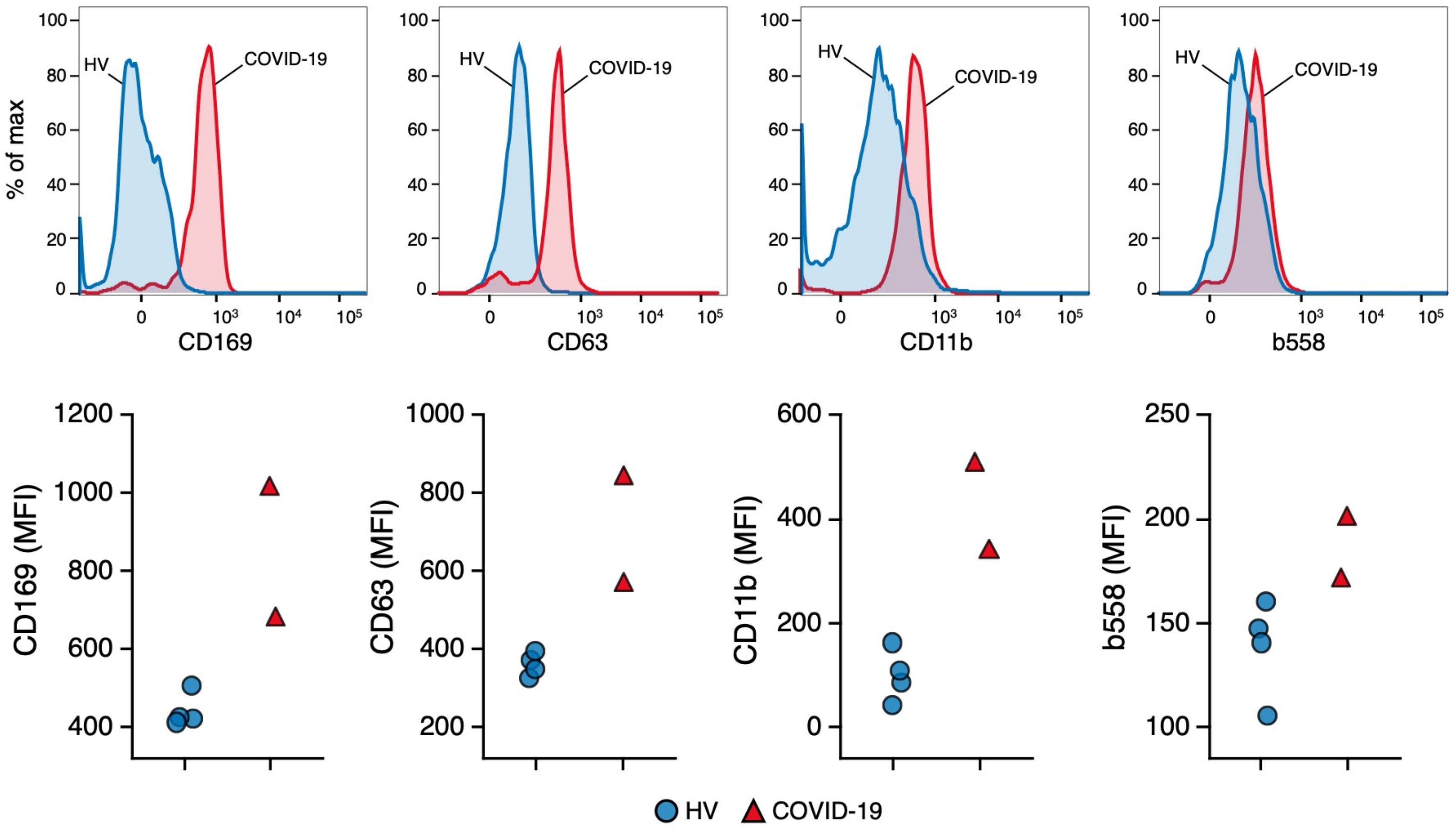

Supplementary Figure 6. CD14⁺ monocytes exhibit an activation phenotype in peripheral blood of COVID-19 patients. Shown are representative histogram plots and summary data of the MFI of surface expression of CD169, CD63, CD11b and b558 in CD14⁺ monocytes from hospitalized hypoxemic COVID-19 patients (n = 2) relative to healthy volunteers (HV; n = 4).

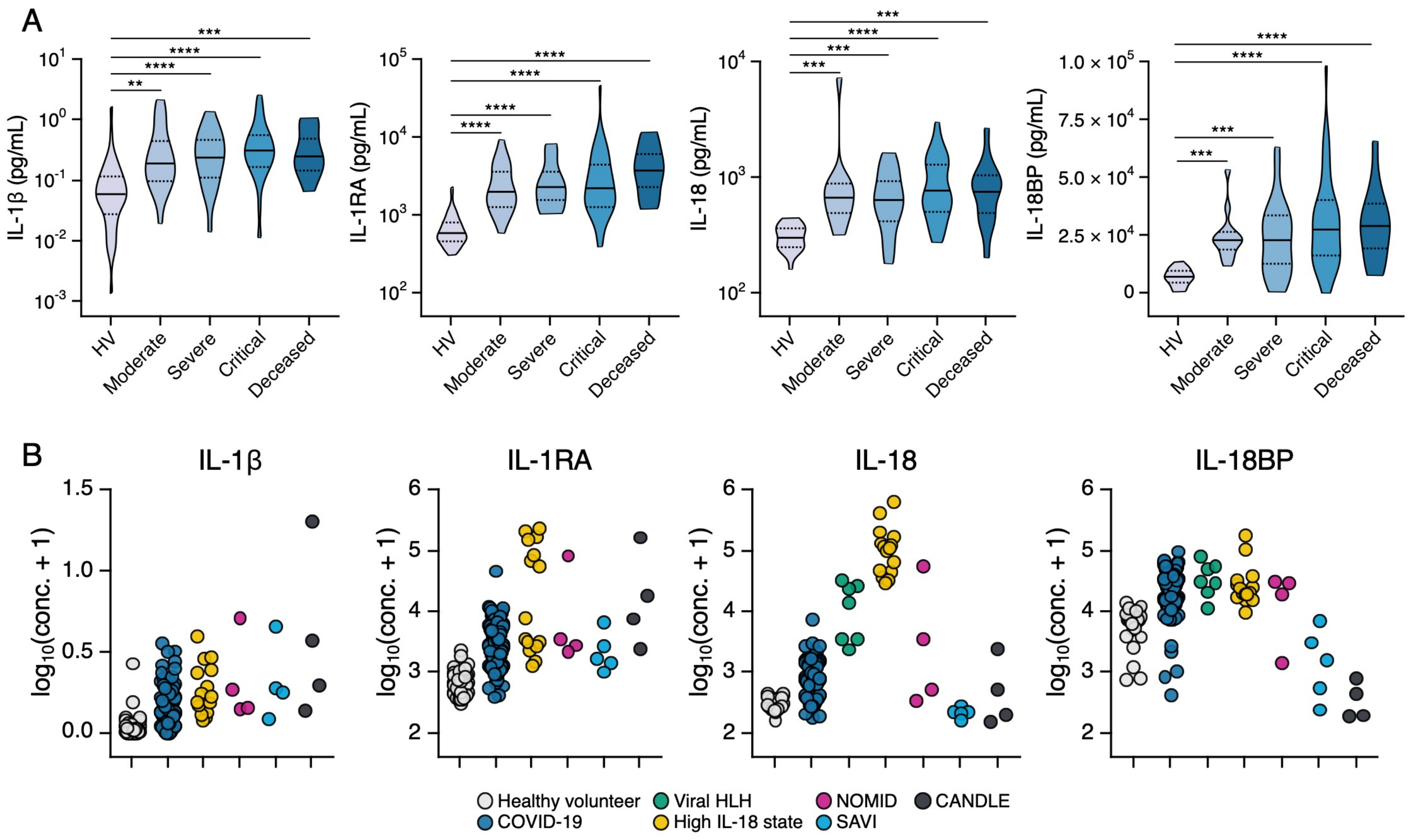

Supplementary Figure 7. **Inflammasome activation in COVID-19 patients. (A)** Shown are levels of IL-1β, IL-1RA, IL-18 and IL-18BP in peripheral blood of COVID-19 patients with various severity groups (n = 103-119 depending on the biomarker) relative to healthy volunteers (HV; n = 24-60 depending on the biomarker). Groups were compared by Kruskal-Wallis test. When *P*<0.05, pairwise comparisons were made using Dunn's test with Benjamini-Hochberg adjustment for

multiple comparisons. *$P<0.05$, **$P<0.01$, ***$P<0.001$, ****$P<0.0001$. **(B)** Shown are levels of IL-1β, IL-1RA, IL-18 and IL-18BP in peripheral blood of COVID-19 patients (n = 103-119 depending on the biomarker) relative to HV (n = 24-60 depending on the biomarker) and patients with the indicated canonical IL-1β- or IL-18-driven autoinflammatory diseases (n = 4-15 depending on the disease as outlined below) or virus-associated hemophagocytic lymphohystiocytosis (HLH). NOMID, Neonatal onset multisystem inflammatory disease; CANDLE; Chronic atypical neutrophilic dermatosis with lipodystrophy and elevated temperature; SAVI; STING-associated vasculopathy with onset in infancy; The 15 High IL-18 state patients consist of 8 patients with IL-18 PAP-MAS, 3 patients with NLRC4-MAS, 2 patients with CDC42-mediated autoinflammatory disease, 1 patient with SJIA and MAS, and 1 patient with adult-onset Still's disease. MAS, macrophage activation syndrome; SJIA, systemic juvenile idiopathic arthritis; IL18 PAP-MAS; IL-18-mediated pulmonary alveolar proteinosis and macrophage activation syndrome; NLRC4, NLR family CARD domain-containing protein 4; CDC42, Cell division control protein 42 homolog.

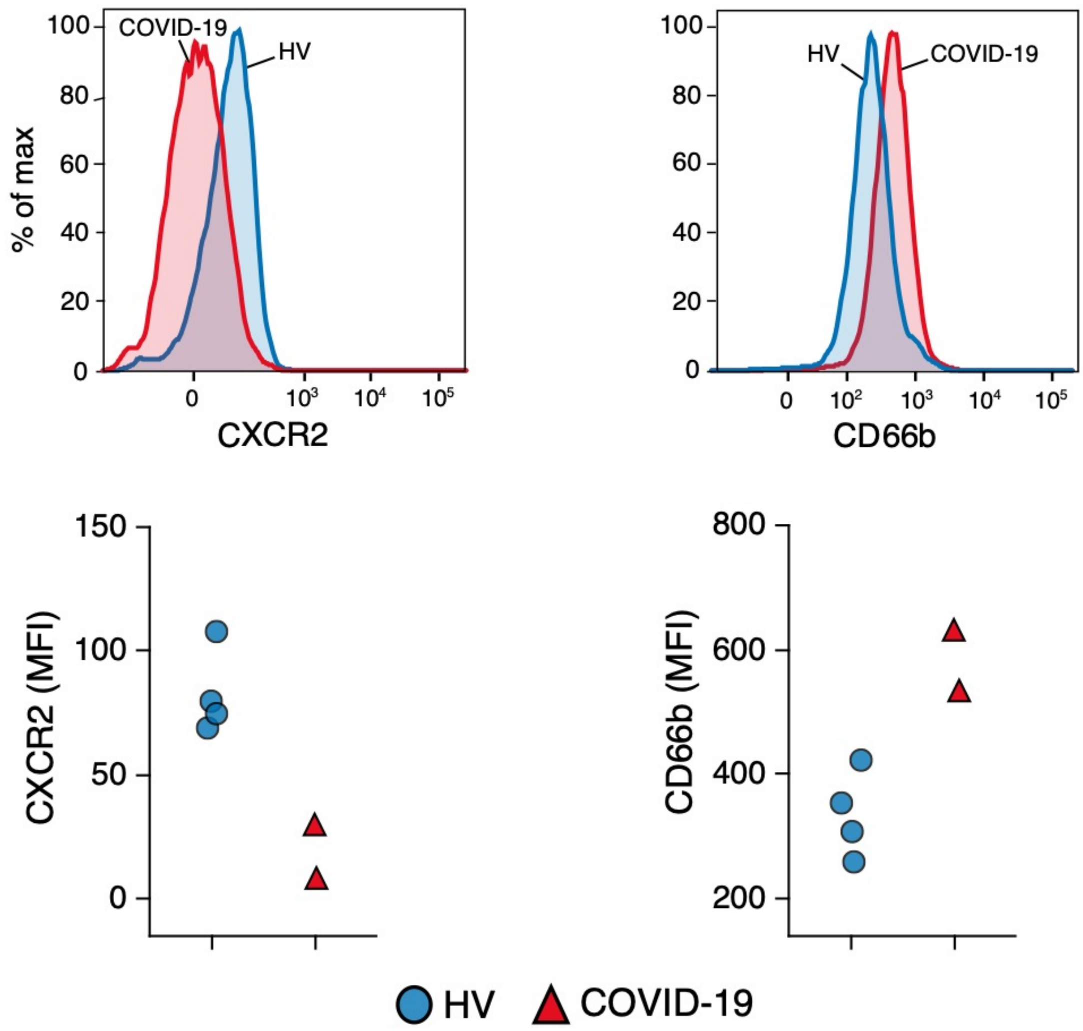

Supplementary Figure 8. Neutrophils exhibit an activation phenotype in peripheral blood of COVID-19 patients. Shown are representative histogram plots and summary data of the MFI of surface expression of CXCR2 and CD66b in neutrophils from hospitalized hypoxemic COVID-19 patients (n = 2) relative to healthy volunteers (HV; n = 5).

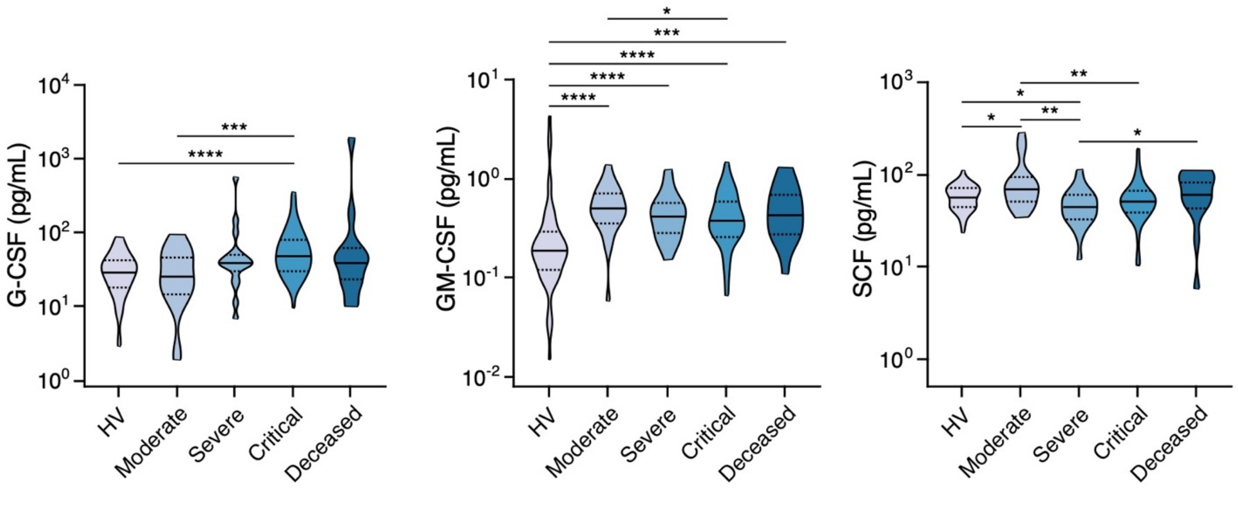

Supplementary Figure 9. **GM-CSF and SCF are differentially increased in COVID-19 patients with different severity.** Shown are levels of G-CSF, GM-CSF and SCF in peripheral blood of COVID-19 patients with various severity groups (n = 119) relative to healthy volunteers (HV; n = 45-60 depending on the biomarker). Groups were compared by Kruskal-Wallis test. When *P*<0.05, pairwise comparisons were made using Dunn's test with Benjamini-Hochberg adjustment for multiple comparisons. *P<0.05, **P<0.01, ***P<0.001, ****P<0.0001.

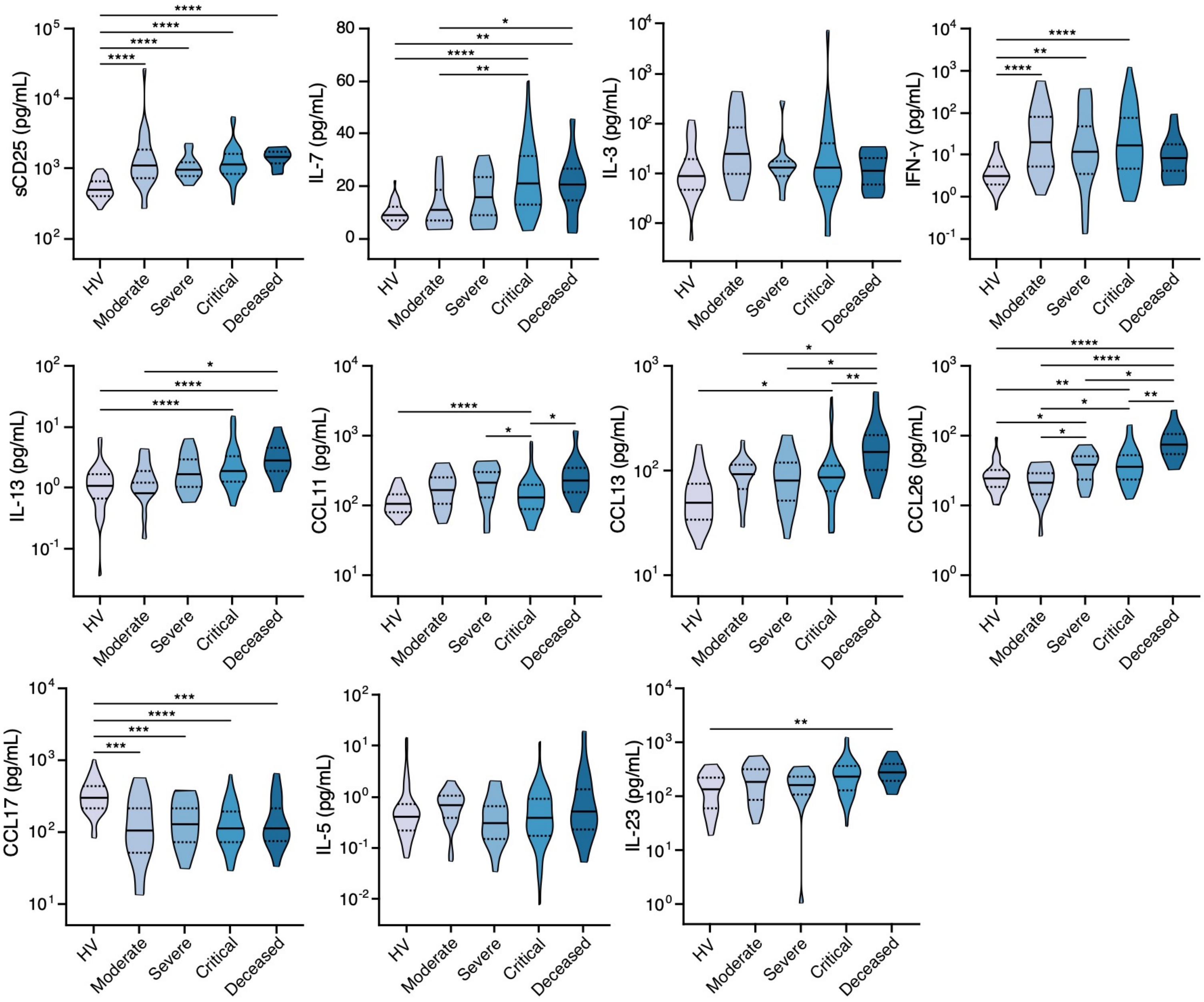
sCD25 (pg/mL)
IL-7 (pg/mL)
IL-3 (pg/mL)
IFN-γ (pg/mL)
IL-13 (pg/mL)
CCL11 (pg/mL)
CCL13 (pg/mL)
CCL26 (pg/mL)
CCL17 (pg/mL)
IL-5 (pg/mL)
IL-23 (pg/mL)
HV
Moderate
Severe
Critical
Deceased

Supplementary Figure 10. **Levels of additional biomarkers associated with T cell activation and polarization in COVID-19 patients.** Shown are levels of sCD25, IL-7, IL-3, IFN-γ, IL-13, CCL11, CCL13, CCL26, CCL17, IL-5 and IL-23 in peripheral blood of COVID-19 patients with various severity groups (n = 94-119 depending on the biomarker) relative to healthy volunteers (HV; n = 45-60 depending on the biomarker). Groups were compared by Kruskal-Wallis test. When $P<0.05$, pairwise comparisons were made using Dunn's test with Benjamini-Hochberg adjustment for multiple comparisons. *$P<0.05$, **$P<0.01$, ***$P<0.001$, ****$P<0.0001$.

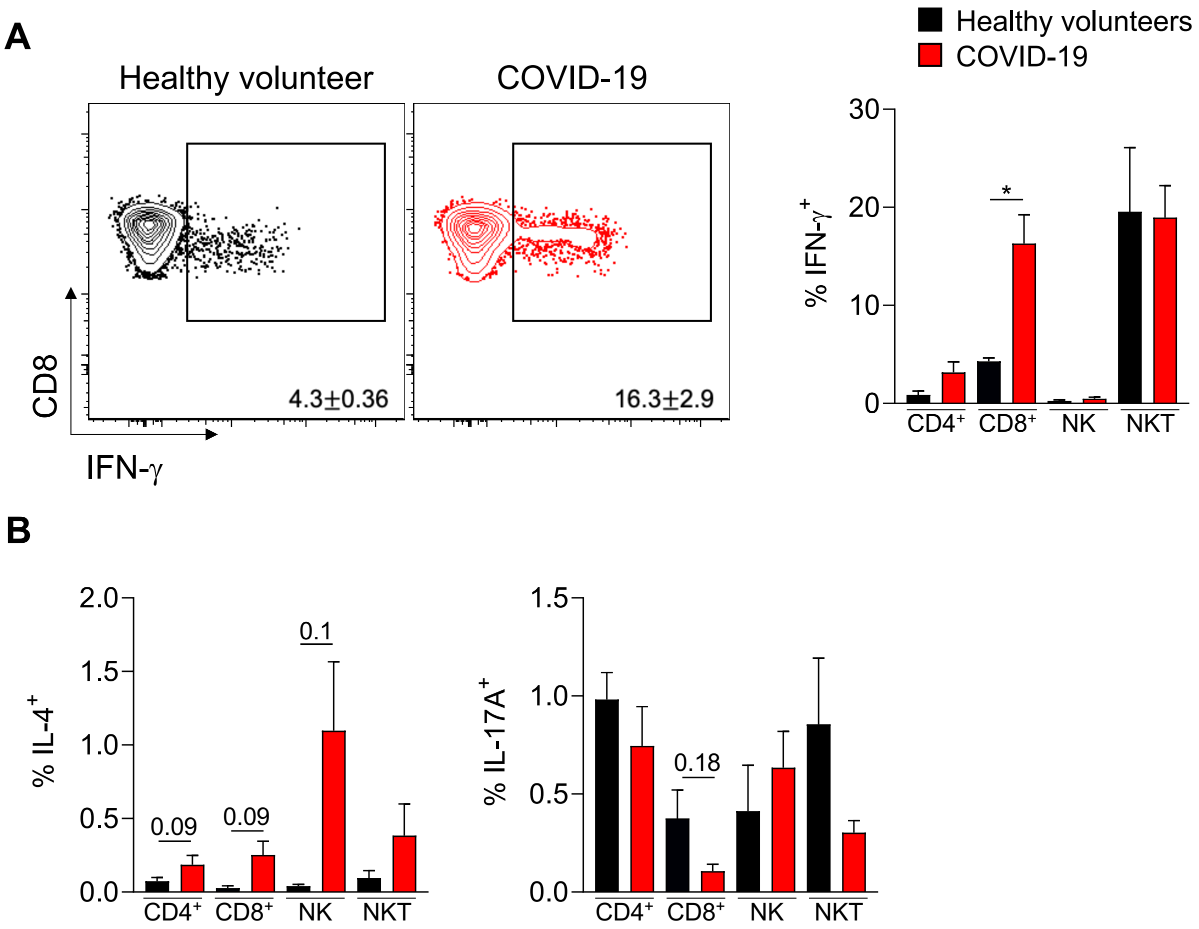

Supplementary Figure 11. CD8⁺ T cells of patients with COVID-19 exhibit enhanced IFN-γ production. (A) Left panel depicts representative contour plots of IFN-γ production within $CD8^+$ T cells in unstimulated whole blood of patients with COVID-19 (red) and healthy volunteers (black). Right panel depicts summary data of frequencies of IFN-γ producing cells within the indicated lymphoid cell subsets in patients with COVID-19 (n = 4) and healthy volunteers (n = 6). All quantitative data represent mean ± standard error of the mean. *P* value was calculated using an unpaired t-test with Welch's correction. **P*<0.05. **(B)** Summary data showing no significant differences in the percent of the indicated $IL\text{-}4^+$ or $IL\text{-}17A^+$ lymphoid cell subsets from COVID-19 patients compared to healthy volunteers.

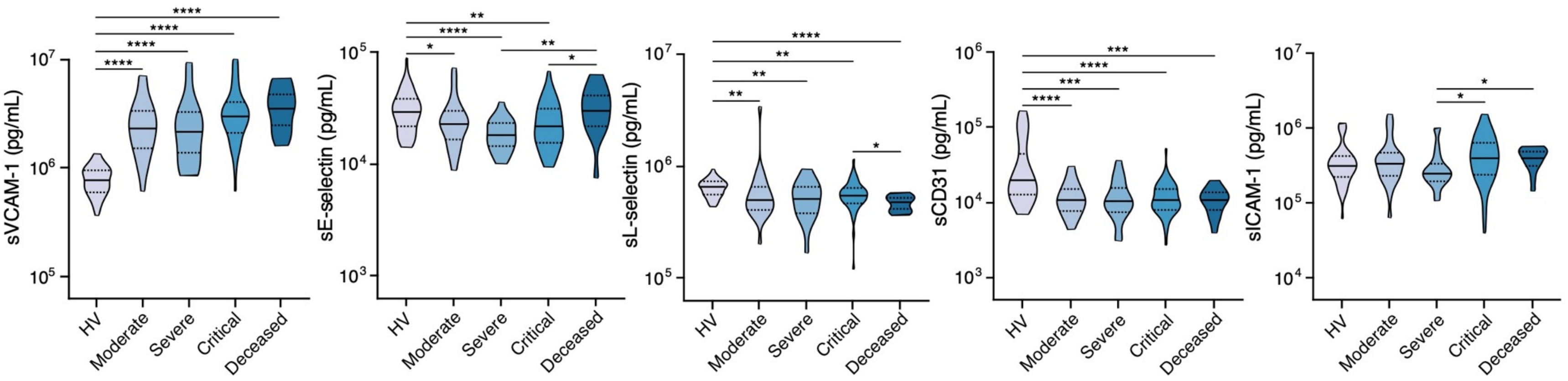

Supplementary Figure 12. **Levels of additional biomarkers associated with endothelial function in COVID-19 patients.** Shown are levels of sVCAM-1, sE-selectin, sL-selectin, sCD31 and sICAM-1 in peripheral blood of COVID-19 patients with various severity groups (n = 94-119 depending on the biomarker) relative to healthy volunteers (HV; n = 45-60 depending on the biomarker). Groups were compared by Kruskal-Wallis test. When *P*<0.05, pairwise comparisons were made using Dunn's test with Benjamini-Hochberg adjustment for multiple comparisons. **P*<0.05, ***P*<0.01, ****P*<0.001, *****P*<0.0001.

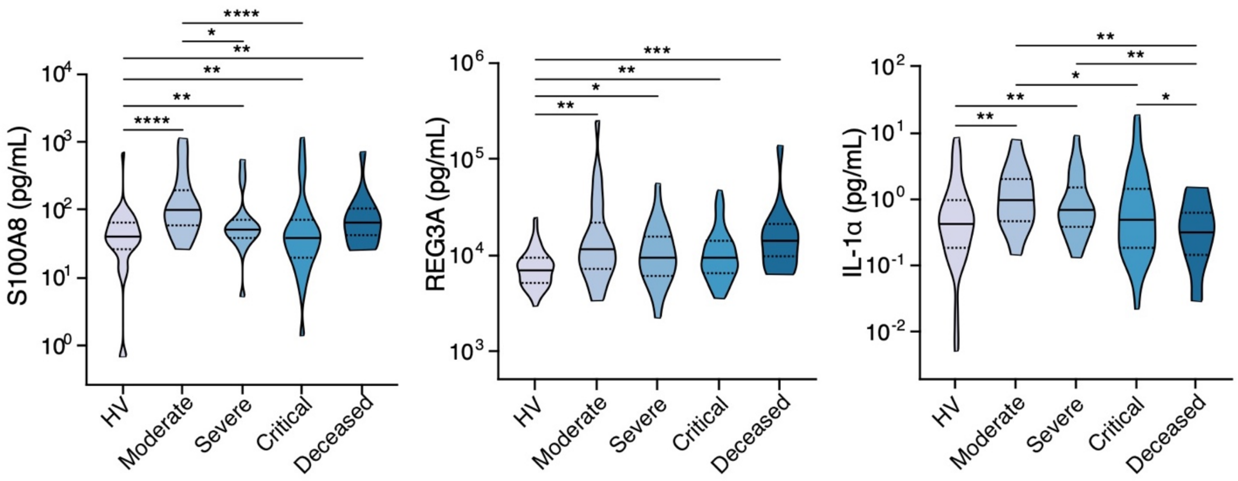

Supplementary Figure 13. **Levels of epithelial cell-associated biomarkers in COVID-19 patients.** Shown are levels of S100A8, REG3A and IL-1α in peripheral blood of COVID-19 patients with various severity groups (n = 119) relative to healthy volunteers (HV; n = 60). Groups were compared by Kruskal-Wallis test. When *P*<0.05, pairwise comparisons were made using Dunn's test with Benjamini-Hochberg adjustment for multiple comparisons. **P*<0.05, ***P*<0.01, ****P*<0.001, *****P*<0.0001.

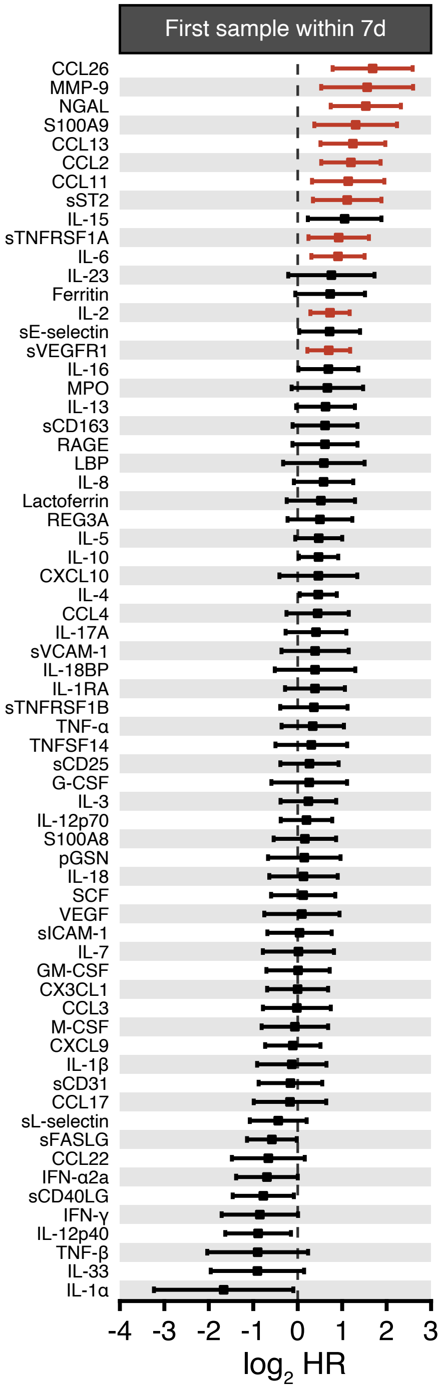

Supplementary Figure 14. Biomarkers associated with mortality in COVID-19 patients in univariable analysis. Shown are forest plots and HRs of all 66 tested biomarkers and their association with mortality during COVID-19 by univariable analysis in the 119 patients in whom the first sample was collected within the first week of hospital admission. For biomarkers significantly associated with mortality (i.e., q-value <0.025), HR confidence intervals are shown in red.

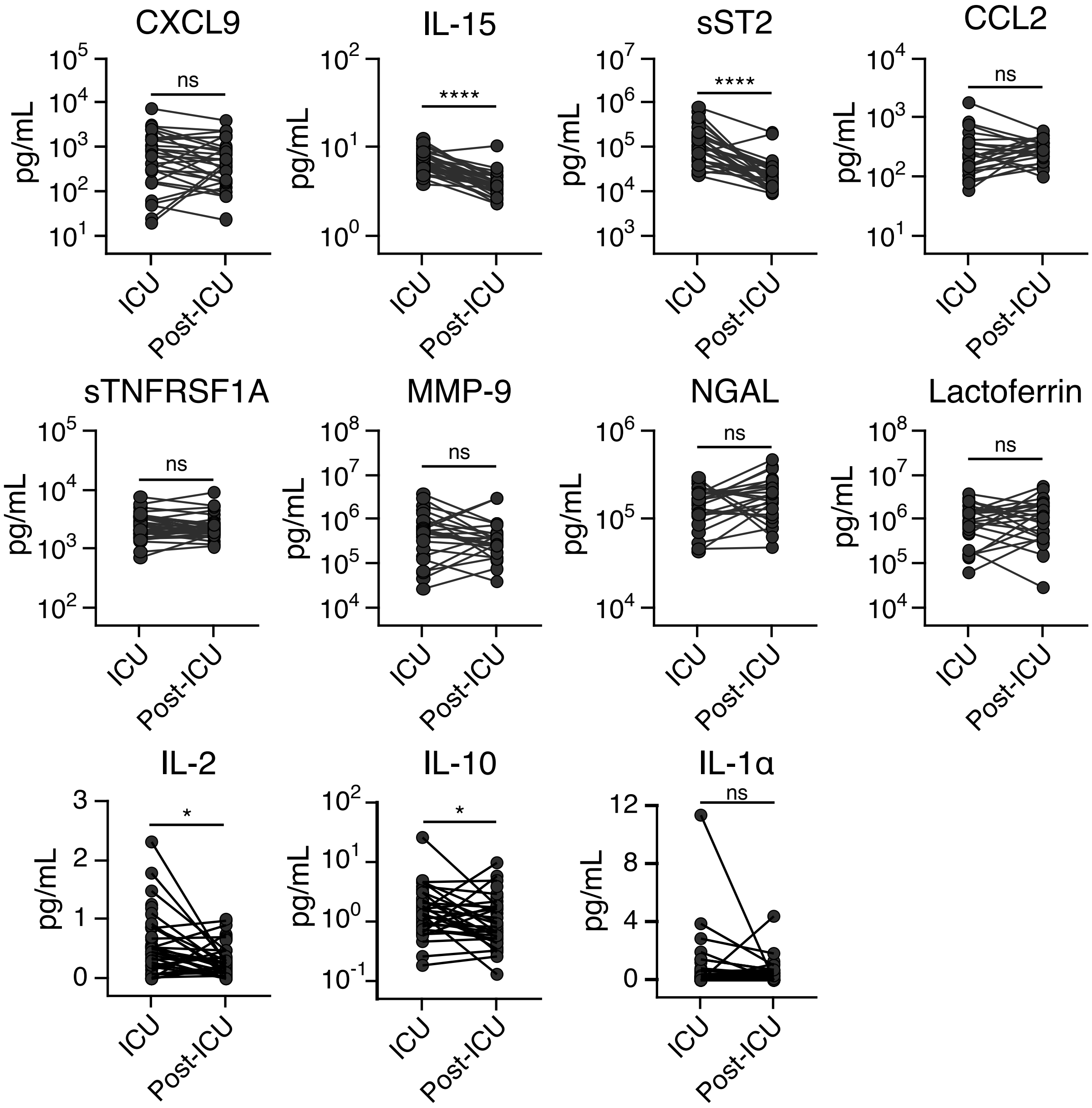
CXCL9
IL-15
sST2
CCL2
sTNFRSF1A
MMP-9
NGAL
Lactoferrin
IL-2
IL-10
IL-1α
pg/mL
ICU
Post-ICU
ns
****
*

Supplementary Figure 15. **Changes in biomarker concentration during recovery from COVID-19 in critically ill ICU patients.** Shown are levels of CXCL9, IL-15, sST2, CCL2, sTNFRSF1A, MMP-9, NGAL, lactoferrin, IL-2, IL-10, and IL-1α in the same patients during ICU admission and later on after patient recovery. n = 24-30 depending on the biomarker. Samples collected during ICU course were compared to those obtained post-ICU discharge by a paired Mann-Whitney U test. **P*<0.05, *****P*<0.0001. ICU, intensive care unit; ns, not significant.

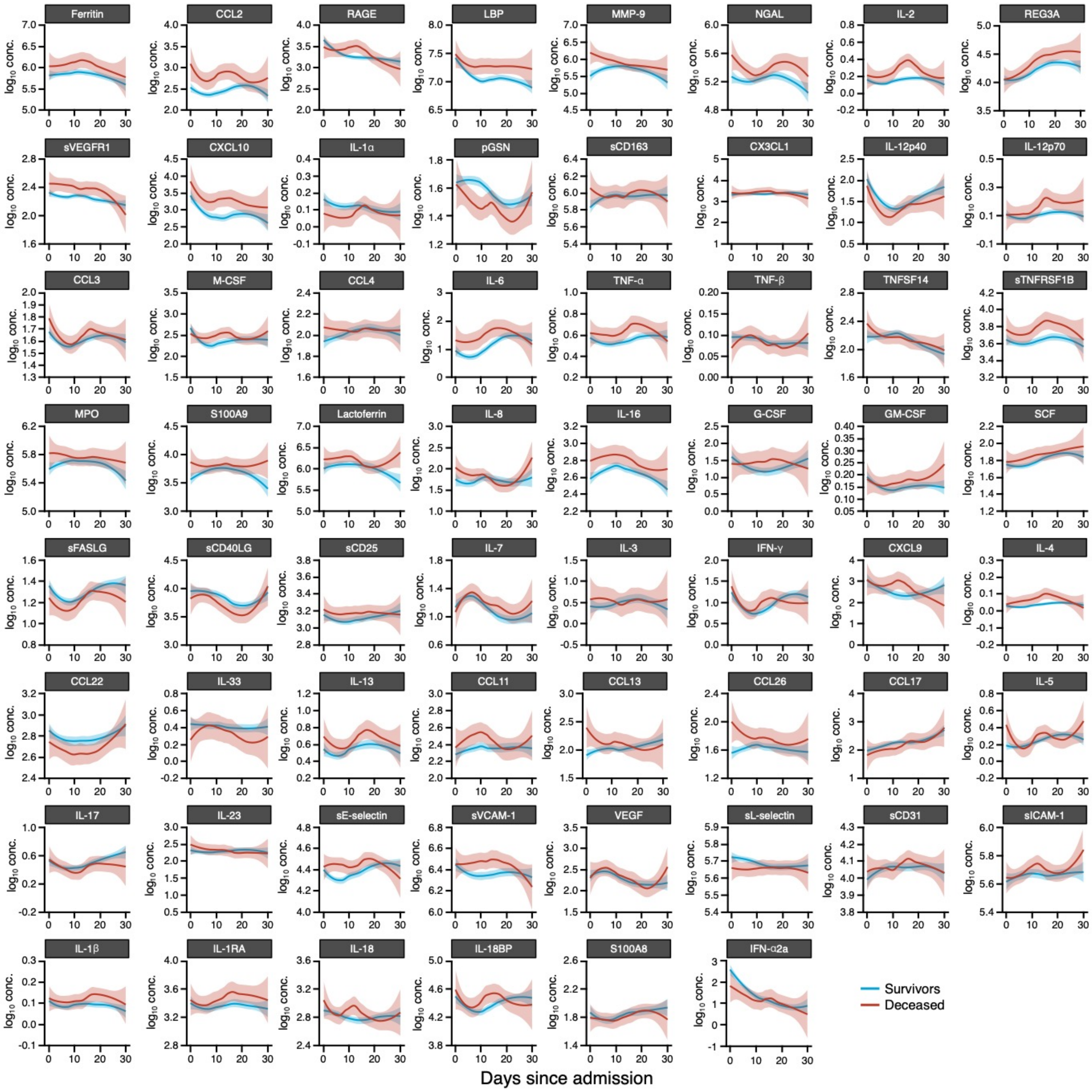

Supplementary Figure 16. **Longitudinal trajectory of biomarkers in survivors and those who died from COVID-19 throughout the entire hospitalization.** Shown are loess smoothed means with 95% confidence intervals (shaded intervals) of biomarker concentration throughout the course of hospitalization in patients with COVID-19 who survived or succumbed to the infection (n = 149-175 patients, 418-609 samples depending on the biomarker). All biomarker concentrations are in pg/mL with the exception of IFN-α2a that are in fg/mL and pGSN that is in μg/mL.

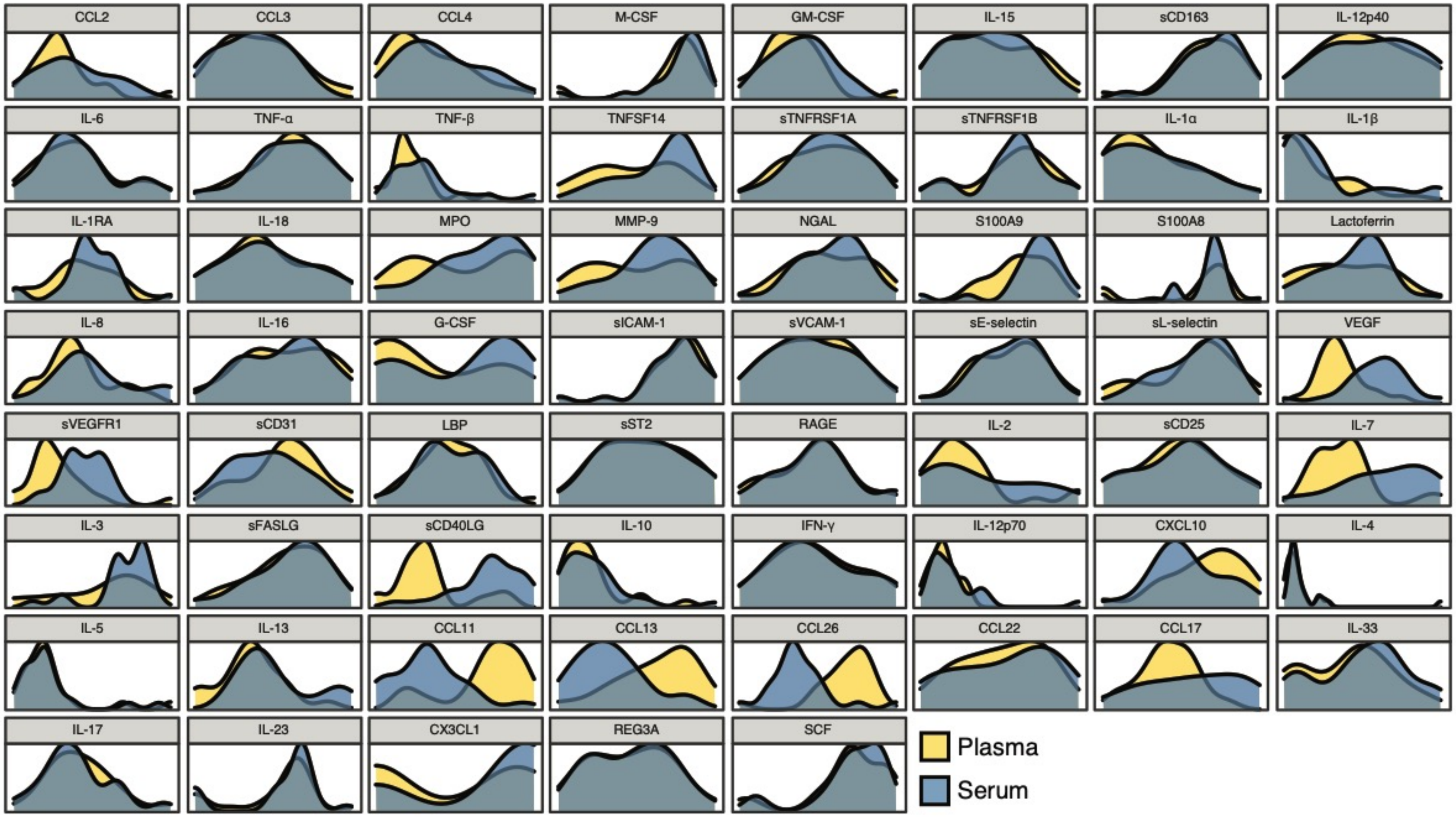

Supplementary Figure 17. Distribution of biomarker concentrations in serum versus plasma samples in COVID-19 patients. Shown are density plots depicting the distribution of biomarker concentrations from 22 paired serum and plasma collections (i.e. both serum and plasma collected on the same day from an individual patient).

Supplementary Table 1. Clinical characteristics of the COVID-19 patients included in our study.

Characteristic	All patients (n = 175)	First sample within 7 days of admission (n = 119)
**Male**	132 (75.9%)	86 (72.3%)
**Age**	60 (51-69) years	57 (49-69) years
**Site in Italy**		
Brescia	153 (87.4%)	101 (84.9%)
Monza	17 (9.7%)	16 (13.4%)
Pavia	5 (2.9%)	2 (1.7%)
**Comorbidities**[#]		
None	32/172 (18.6%)	22/117 (18.8%)
Hypertension	64/172 (37.2%)	42/117 (35.9%)
Diabetes mellitus	36/172 (20.9%)	23/117 (19.7%)
Cardiovascular disease*	32/172 (18.6%)	21/117 (17.9%)
Autoimmune disease	29/172 (16.9%)	20/117 (17.1%)
Neurologic disease	25/172 (14.5%)	17/117 (14.5%)
Hematologic malignancy	20/172 (11.6%)	14/117 (12.0%)
Obesity (BMI >30)	17/172 (9.9%)	12/117 (10.3%)
Chronic respiratory disease	15/172 (8.7%)	5/117 (4.3%)
Thyroid disease	15/172 (8.7%)	8/117 (6.8%)
Solid organ malignancy	15/172 (8.7%)	10/117 (8.5%)
Chronic kidney disease	14/172 (8.1%)	12/117 (10.3%)
Hyperlipidemia	13/172 (7.6%)	8/117 (6.8%)
Chronic gastrointestinal disease**	13/172 (7.6%)	8/117 (6.8%)
Psychiatric illness	9/172 (5.2%)	6/117 (5.1%)
Chronic liver disease	8/172 (4.7%)	4/117 (3.4%)
HIV positive	6/172 (3.5%)	5/117 (4.3%)
Asthma	5/172 (2.9%)	3/117 (2.6%)
Benign hematologic disease	5/172 (2.9%)	4/117 (3.4%)
Solid organ transplantation	4/172 (2.3%)	3/117 (2.6%)
**Clinical features/medical interventions**		
Duration of symptoms prior to hospitalization	7 (5-10) days	7 (5-11) days
Supplemental oxygen required	157/175 (89.7%)	97/119 (81.5%)
Invasive mechanical ventilation	69/175 (39.4%)	30/119 (25.2%)
Anticoagulation^	105/160 (65.6%)	80/117 (68.4%)

Corticosteroids^	93/160 (58.1%)	68/117 (58.1%)
Tocilizumab^	40/160 (25.0%)	16/117 (13.7%)
Canakinumab^	7/160 (4.4%)	7/117 (6.0%)
**ICU care**	70/175 (40.0%)	33/119 (27.7%)
**Severity**		
Mild/moderate	30/175 (17.2%)	26/119 (21.8%)
Severe	23/175 (13.1%)	20/119 (16.8%)
Critical[@]	89/175 (50.9%)	58/119 (48.7%)
Deceased	33/175 (18.9%)	15/119 (12.6%)

Data are n (%) or median (interquartile range) unless stated otherwise. Percentages refer to the number of patients with feature divided by the number of evaluable patients
[#]Data on comorbidities were available in 172 of 175 total patients and 117 of 119 patients who had the first sample collected within 7 days of hospital admission
* Not including hypertension
** Not including hepatobiliary diseases
^Data on medical therapies listed were available in 160 of 175 total patients and 117 of 119 patients who had the first sample collected within 7 days of hospital admission
[@]A total of 122 patients with critical COVID-19 were enrolled, of which 89 survived the infection and 33 succumbed to COVID-19.
BMI, body mass index; COPD, chronic obstructive pulmonary disease; ICU, intensive care unit.

Supplementary Table 2. Summary data depicting the association of clinical factors and laboratory tests with mortality in univariable analyses.

Variable	HR (95% CI)	*P* value
**Demographics**		
Age ≥65 years	3.96 (1.76-8.89)	0.0009
Male sex	1.86 (0.65-5.34)	0.25
**Comorbidities**		
None	0.64 (0.19-2.10)	0.46
Hypertension	0.79 (0.38-1.67)	0.54
Heart disease	0.90 (0.37-2.22)	0.83
Congestive heart failure	0.98 (0.30-3.23)	0.97
Arrhythmia	1.05 (0.32-3.48)	0.93
Obesity (BMI >30)	0.97 (0.29-3.19)	0.96
Hyperlipidemia	2.30 (0.88-6.02)	0.089
Diabetes mellitus	2.54 (1.23-5.29)	0.012
Chronic respiratory disease	0.93 (0.28-3.07)	0.9
Chronic kidney disease	0.68 (0.16-2.88)	0.61
Chronic liver disease	1.36 (0.32-5.72)	0.67
Gastrointestinal illness	1.72 (0.52-5.67)	0.37
Autoimmune disease	0.68 (0.24-1.95)	0.47
Solid organ malignancy	1.13 (0.34-3.72)	0.84
Hematologic malignancy	1.31 (0.50-3.43)	0.58
**Clinical management**		
ICU care	2.21 (1.01-4.85)	0.048
Intubation	2.65 (1.17-5.99)	0.019
Anticoagulation	0.35 (0.16-0.77)	0.009
Corticosteroids	0.69 (0.31-1.51)	0.35
Tocilizumab	0.36 (0.12-1.05)	0.062
Canakinumab	0.79 (0.11-5.83)	0.82
**Clinical laboratory tests**		
Absolute neutrophil count	1.08 (0.71-1.63)	0.73
Absolute lymphocyte count	0.39 (0.19-0.79)	0.009
NLR	1.62 (1.04-2.53)	0.032
Absolute monocyte count	0.97 (0.64-1.48)	0.89
Absolute eosinophil count	0.56 (0.13-2.33)	0.42
Absolute basophil count	0.86 (0.52-1.40)	0.54
Platelet count	0.77 (0.51-1.16)	0.21

Hemoglobin	0.82 (0.55-1.22)	0.33
BUN	1.21 (0.75-1.94)	0.44
Creatinine	1.12 (0.81-1.55)	0.48
Troponin	1.09 (0.70-1.69)	0.7
CRP	1.28 (0.78-2.09)	0.33
LDH	1.16 (0.77-1.76)	0.48
PT	0.70 (0.36-1.37)	0.3
PTT	0.62 (0.35-1.10)	0.1
D-dimer	1.12 (0.74-1.69)	0.61
Fibrinogen	1.28 (0.81-2.03)	0.29

BMI, body mass index; BUN, blood urea nitrogen; CRP, C-reactive protein; ICU, intensive care unit; LDH, lactate dehydrogenase; NLR, neutrophil to lymphocyte ratio; PT, prothrombin time; PTT, partial thromboplastin time

Supplementary Table 3. Summary data of the concentrations of all 66 tested biomarkers in COVID-19 patients of different severity groups and healthy volunteers.

Submitted as a separate Excel file.

Supplementary Table 4. Summary data of all biomarkers and their association with mortality in three mathematical models of univariable and multivariable analyses.

Submitted as a separate Excel file.

Supplementary Table 5. Evaluation of clinical factors that affect the concentration of immune-based biomarkers and laboratory tests.

Submitted as a separate Excel file.

Supplemental Table 6. Association between the longitudinal trajectory of biomarkers and the risk of death after COVID-19. Shown are the posterior median HR and 95% credible intervals (CrI), along with q-values for the posterior probability that the association was positive or negative. Biomarkers with q-values < 0.025 are highlighted in red.

	Longitudinal model Adjusted* (n = 175)		
**Biomarker**	**aHR (95% CrI)**	**q value, lower***	**q value, upper**
IL-15	14.10 (4.80-45.50)	1.000	0.001
IL-2	13.80 (3.70-56.80)	1.000	0.001
CCL2	5.70 (2.30-16.90)	1.000	0.001
sST2	3.00 (1.70-5.60)	1.000	0.001
NGAL	5.80 (2.00-21.20)	1.000	0.007
sTNFRSF1A	3.00 (1.50-6.10)	1.000	0.012
CXCL9	4.40 (1.50-16.60)	1.000	0.017
MMP-9	3.10 (1.40-8.40)	1.000	0.017
IL-10	2.00 (1.30-3.10)	1.000	0.018
Lactoferrin	4.40 (1.50-18.60)	1.000	0.023
LBP	3.20 (1.30-9.70)	1.000	0.029
sVEGFR1	2.60 (1.30-5.00)	1.000	0.029
IL-13	5.70 (1.30-24.30)	1.000	0.043
MPO	5.20 (1.30-24.20)	1.000	0.043
IL-16	2.70 (1.20-6.50)	1.000	0.043
Ferritin	1.90 (1.10-3.30)	1.000	0.043
IL-12p70	6.20 (1.30-28.40)	1.000	0.044
IL-6	1.40 (1.00-1.80)	1.000	0.044
TNF-α	4.70 (1.10-20.50)	1.000	0.045
S100A9	3.70 (1.20-14.30)	1.000	0.045
CCL26	2.90 (1.20-8.10)	1.000	0.045
RAGE	2.70 (1.10-6.80)	1.000	0.045
REG3A	1.90 (1.10-3.20)	1.000	0.045
CXCL10	2.10 (1.00-6.80)	1.000	0.047
CX3CL1	1.70 (1.00-3.80)	1.000	0.048
sE-selectin	2.70 (1.00-7.10)	1.000	0.055
G-CSF	1.60 (1.00-2.90)	1.000	0.095
IL-1RA	2.00 (0.90-4.60)	1.000	0.129
sCD25	1.60 (0.90-2.60)	1.000	0.132
CCL13	3.00 (0.70-12.70)	1.000	0.133

IL-4	5.00 (0.60-36.70)	1.000	0.136
CCL11	2.50 (0.70-8.70)	1.000	0.136
SCF	2.00 (0.80-5.10)	1.000	0.150
IL-23	1.20 (0.90-1.50)	1.000	0.179
sTNFRSF1B	1.60 (0.80-3.20)	1.000	0.219
sCD163	1.40 (0.80-2.30)	1.000	0.225
CCL4	1.90 (0.60-5.90)	1.000	0.236
TNFSF14	1.60 (0.60-5.00)	1.000	0.306
IL-8	1.60 (0.60-4.30)	1.000	0.306
IL-18BP	0.60 (0.20-1.50)	1.000	0.306
sVCAM-1	1.40 (0.60-3.40)	1.000	0.350
VEGF	1.30 (0.50-3.80)	1.000	0.441
IL-5	1.40 (0.40-4.90)	1.000	0.462
IL-7	1.40 (0.30-7.00)	1.000	0.498
CCL3	1.40 (0.30-7.00)	1.000	0.503
GM-CSF	1.50 (0.20-10.60)	1.000	0.510
sCD31	1.30 (0.30-6.80)	1.000	0.510
IFN-γ	1.00 (0.40-2.40)	1.000	0.636
M-CSF	1.00 (0.70-1.50)	1.000	0.695
sICAM-1	1.00 (0.50-2.00)	1.000	0.695
IL-18	2.50 (0.30-19.30)	1.000	0.708
IL-3	1.00 (0.70-1.30)	1.000	0.740
IL-1β	0.80 (0.10-4.80)	1.000	0.740
TNF-β	0.70 (0.10-6.20)	1.000	0.759
S100A8	0.90 (0.60-1.40)	1.000	0.771
sL-selectin	0.70 (0.20-2.00)	1.000	0.856
IL-17A	0.90 (0.30-2.90)	1.000	0.969
IFN-α2a	0.80 (0.50-1.10)	0.810	0.991
sFASLG	0.60 (0.30-1.40)	0.810	0.991
CCL17	0.50 (0.10-1.30)	0.810	0.991
pGSN	0.40 (0.10-1.60)	0.810	0.991
sCD40LG	0.30 (0.10-1.50)	0.810	0.991
IL-12p40	0.50 (0.30-0.90)	0.189	1.000
IL-33	0.40 (0.20-1.10)	0.593	1.000
CCL22	0.40 (0.10-1.00)	0.505	1.000
IL-1α	0.20 (0.10-0.50)	0.014	1.000

* Adjusted for time to sampling, age, chronic kidney disease, and use of immunosuppressive medications prior to sample collection

Supplementary Table 7. Age and gender characteristics of HV included in this study.

Age (years)	Gender
33.1	Male
33.4	Female
48.9	Male
31.3	Male
48.1	Male
49.8	Female
55.0	Female
51.7	Female
39.7	Female
57.1	Male
40.3	Female
25.2	Male
27.8	Male
50.0	Male
30.7	Male
55.9	Male
44.1	Female
56.0	Female
53.3	Female
39.0	Male
39.7	Female
27.1	Male
37.0	Male
64.9	Male
45.0	Female
60.9	Male
32.1	Female
48.9	Male
32.0	Female
51.3	Female
46.2	Male
46.7	Female
32.5	Male
57.9	Male
40.1	Female
28.0	Male

36.4	Male
56.0	Male
27.2	Male
50.2	Female
58.6	Female
37.5	Female
54.4	Male

Data on gender and age were available for 58 of 60 HV.

Supplementary Table 8. Summary of statistical models describing the association between time to death and biomarker concentration.

**Model 1:** Cox proportional hazards for risk of death versus biomarker concentration.<br>**Data:** first sample.<br>**Additional covariates:** time from admission to sample collection.
**Model 2:** Cox proportional hazards for risk of death versus biomarker concentration.<br>**Data:** first sample with missing co-variate data imputed.<br>**Additional covariates:** time from admission to sample collection, age, history of chronic kidney disease, receipt of immunomodulatory medications.
**Model 3:** Bayesian shared parameter joint model for longitudinal biomarker trajectory and risk of death versus expected biomarker concentration.<br>**Longitudinal submodel:** mixed effects generalized linear model for log-biomarker concentration versus time from admission with subject-specific intercepts.<br>**Survival submodel:** proportional hazards model for risk of death versus expected biomarker concentration from the longitudinal submodel.<br>**Data:** All samples with missing biomarker and covariate data imputed.<br>**Additional covariates:**<br>*Longitudinal submodel:* time from admission to sample collection.<br>*Survival submodel:* age, history of chronic kidney disease, receipt of immunomodulatory medications.

Supplementary Table 9. Sample-level distribution of missingness of clinical characteristics and biomarker measurements. The joint distribution of these variables was used to impute the missing values.

	Per sample analysis (n = 609)		Per patient analysis (n = 175)	
Variable	No. with missing data	% with missing data	No. with missing data	% with missing data
Demographics				
Gender	2	0.33	1	0.57
Age	2	0.33	1	0.57
**Comorbidities**			18	10.29
Heart disease	18	2.96	3	1.71
Hypertension	18	2.96	3	1.71
Congestive heart failure	18	2.96	3	1.71
Arrhythmia	18	2.96	3	1.71
Hyperlipidemia	18	2.96	3	1.71
Chronic respiratory disease	18	2.96	3	1.71
Asthma	18	2.96	3	1.71
Chronic obstructive pulmonary disease	18	2.96	3	1.71
Chronic kidney disease	18	2.96	3	1.71
Chronic gastrointestinal disease	18	2.96	3	1.71
Chronic liver disease	18	2.96	3	1.71
Diabetes mellitus	18	2.96	3	1.71
Thyroid disease	18	2.96	3	1.71
Obesity (BMI >30)	18	2.96	3	1.71
Solid organ malignancy	18	2.96	3	1.71
Hematologic malignancy	18	2.96	3	1.71
Autoimmune disease	18	2.96	3	1.71
Chronic skin condition	18	2.96	3	1.71
Neurologic disorder	18	2.96	3	1.71
Psychiatric illness	18	2.96	3	1.71
Benign hematologic condition	18	2.96	3	1.71
Human immunodeficiency virus	18	2.96	3	1.71
Other immunodeficiency	18	2.96	3	1.71

Solid organ transplantation	18	2.96	3	1.71
**Timing of major events relative to hospital admission**				
Days from self-reported symptom onset to hospital admission	57	9.36	29	16.57
Days from admission to sample collection	0	0	109	62.29
Days from hospital admission to discharge/death	0	0	0	0
**Clinical management**				
ICU care	0	0	0	0
Supplemental oxygen	18	2.96	6	3.43
**Treatment**				
Corticosteroids	70	11.49	21	12
Tocilizumab	58	9.52	18	10.29
Canakinumab	58	9.52	18	10.29

BMI, body mass index; ICU, intensive care unit

Supplementary Table 10. Patient-level distribution of missingness of clinical characteristics. The joint distribution of clinical characteristics and longitudinal biomarker values were used to impute the missing values.

Biomarker	No. of samples with available data	% of samples with available data	No. of samples with missing data	% of samples with missing data
CCL11	418	68.64%	191	31.36%
CCL13	418	68.64%	191	31.36%
CCL17	417	68.47%	192	31.53%
CCL2	418	68.64%	191	31.36%
CCL22	609	100.00%	0	0.00%
CCL26	418	68.64%	191	31.36%
CCL3	609	100.00%	0	0.00%
CCL4	609	100.00%	0	0.00%
sCD163	609	100.00%	0	0.00%
sCD25	609	100.00%	0	0.00%
sCD31	609	100.00%	0	0.00%
sCD40LG	418	68.64%	191	31.36%
CX3CL1	609	100.00%	0	0.00%
CXCL10	418	68.64%	191	31.36%
CXCL9	609	100.00%	0	0.00%
sE-selectin	609	100.00%	0	0.00%
sFASLG	609	100.00%	0	0.00%
Ferritin	609	100.00%	0	0.00%
G-CSF	609	100.00%	0	0.00%
pGSN	466	76.52%	143	23.48%
GM-CSF	609	100.00%	0	0.00%
sICAM-1	609	100.00%	0	0.00%
IFN-α2a	608	99.84%	1	0.16%
IFN-γ	609	100.00%	0	0.00%
IL-10	609	100.00%	0	0.00%
IL-12p40	609	100.00%	0	0.00%
IL-12p70	609	100.00%	0	0.00%
IL-13	418	68.64%	191	31.36%
IL-15	609	100.00%	0	0.00%
IL-16	609	100.00%	0	0.00%
IL-17A	609	100.00%	0	0.00%
IL-18	403	66.17%	206	33.83%
IL-18BP	403	66.17%	206	33.83%

IL-1α	609	100.00%	0	0.00%
IL-1β	609	100.00%	0	0.00%
IL-1RA	608	99.84%	1	0.16%
IL-2	609	100.00%	0	0.00%
IL-23	609	100.00%	0	0.00%
IL-3	609	100.00%	0	0.00%
IL-33	609	100.00%	0	0.00%
IL-4	609	100.00%	0	0.00%
IL-5	609	100.00%	0	0.00%
IL-6	609	100.00%	0	0.00%
IL-7	418	68.64%	191	31.36%
IL-8	418	68.64%	191	31.36%
Lactoferrin	418	68.64%	191	31.36%
LBP	609	100.00%	0	0.00%
sL-selectin	609	100.00%	0	0.00%
M-CSF	609	100.00%	0	0.00%
MMP-9	418	68.64%	191	31.36%
MPO	418	68.64%	191	31.36%
NGAL	418	68.64%	191	31.36%
RAGE	609	100.00%	0	0.00%
REG3A	609	100.00%	0	0.00%
S100A8	609	100.00%	0	0.00%
S100A9	418	68.64%	191	31.36%
SCF	609	100.00%	0	0.00%
sST2	609	100.00%	0	0.00%
TNF-α	609	100.00%	0	0.00%
TNF-β	609	100.00%	0	0.00%
sTNFRSF1A	609	100.00%	0	0.00%
sTNFRSF1B	609	100.00%	0	0.00%
TNFSF14	418	68.64%	191	31.36%
sVCAM-1	609	100.00%	0	0.00%
VEGF	418	68.64%	191	31.36%
sVEGFR1	609	100.00%	0	0.00%

Supplementary Table 11. Genes included in the type I interferon-regulated, NF-κB-regulated and IFN-γ-regulated scores.

Type I interferon-regulated gene score (n = 28)		NF-κB-regulated gene score (n = 11)	IFN-γ-regulated gene score (n = 15)
EPSTI1	*IFI44*	*AICDA*	*AHR*
IFIT1	*IFI44L*	*CCND2*	*B4GALT3*
IFIT2	*IFI6*	*EBI3*	*BMF*
IFIT3	*IFIT5*	*GZMB*	*CXCL9*
ISG15	*LAMP3*	*IFNG*	*DNA2*
LY6E	*MX1*	*MSR1*	*FBXL5*
USP18	*OAS1*	*SELL*	*GPR146*
CXCL10	*OAS2*	*SELP*	*GRP18*
SOCS1	*OAS3*	*TANK*	*IFTIM1*
GBP1	*OASL*	*TLR2*	*PKDCC*
DDX60	*RSAD2*	*XIAP*	*RAPGEF6*
HERC5	*RTP4*		*SELP*
HERC6	*SIGLEC1*		*SLC5A6*
IFI27	*SPATS2L*		*TRIB3*
			TRIM16
